# Multi-kinase inhibitors can associate with heat shock proteins through their NH_2_-termini by which they suppress chaperone function

**DOI:** 10.18632/oncotarget.7349

**Published:** 2016-02-12

**Authors:** Laurence Booth, Brian Shuch, Thomas Albers, Jane L. Roberts, Mehrad Tavallai, Stefan Proniuk, Alexander Zukiwski, Dasheng Wang, Ching-Shih Chen, Don Bottaro, Heath Ecroyd, Iryna O. Lebedyeva, Paul Dent

**Affiliations:** ^1^ Department of Biochemistry and Molecular Biology, Virginia Commonwealth University, Richmond, VA 23298, USA; ^2^ Urologic and Diagnostic Radiology, Yale School of Medicine, New Haven, CT 06520-8058, USA; ^3^ Urologic Oncology Branch, National Cancer Institute, Bethesda, MD 20892, USA; ^4^ Molecular and Translational Science, United States Medicinal Chemistry and Pharmacognosy, School of Pharmacy, The Ohio State University, Columbus, OH 43210, USA; ^5^ Arno Therapeutics, Flemington, NJ 08822, USA; ^6^ School of Biological Sciences and Illawarra Health and Medical Research Institute, University of Wollongong, NSW 2522, Australia; ^7^ Department of Chemistry and Physics, Augusta University, Augusta, GA 30912, USA

**Keywords:** OSU-03012, sorafenib, pazopanib, chaperones, ATPase

## Abstract

We performed proteomic studies using the GRP78 chaperone-inhibitor drug AR-12 (OSU-03012) as bait. Multiple additional chaperone and chaperone-associated proteins were shown to interact with AR-12, including: GRP75, HSP75, BAG2; HSP27; ULK-1; and thioredoxin. AR-12 down-regulated *in situ* immuno-fluorescence detection of ATP binding chaperones using antibodies directed against the NH_2_-termini of the proteins but only weakly reduced detection using antibodies directed against the central and COOH portions of the proteins. Traditional SDS-PAGE and western blotting assessment methods did not exhibit any alterations in chaperone detection. AR-12 altered the sub-cellular distribution of chaperone proteins, abolishing their punctate speckled patterning concomitant with changes in protein co-localization. AR-12 inhibited chaperone ATPase activity, which was enhanced by sildenafil; inhibited chaperone – chaperone and chaperone – client interactions; and docked in silico with the ATPase domains of HSP90 and of HSP70. AR-12 combined with sildenafil in a GRP78 plus HSP27 –dependent fashion to profoundly activate an eIF2α/ATF4/CHOP/Beclin1 pathway in parallel with inactivating mTOR and increasing ATG13 phosphorylation, collectively resulting in formation of punctate toxic autophagosomes. Over-expression of [GRP78 and HSP27] prevented: AR-12 –induced activation of ER stress signaling and maintained mTOR activity; AR-12 –mediated down-regulation of thioredoxin, MCL-1 and c-FLIP-s; and preserved tumor cell viability. Thus the inhibition of chaperone protein functions by AR-12 and by multi-kinase inhibitors very likely explains why these agents have anti-tumor effects in multiple genetically diverse tumor cell types.

## INTRODUCTION

In 2005 the Dent laboratory published studies which strongly argued that the PDK-1 inhibitor OSU-03012 (licensed by Arno Therapeutics from Ohio State University and called AR-12) was *not* primarily acting as a PDK-1 inhibitor to radio-sensitize tumor cells [1, 2]. In 2006 we demonstrated that the primary mechanism by which AR-12 killed tumor cells was via the PKR-like endoplasmic reticulum kinase (PERK)-dependent induction of endoplasmic reticulum stress signaling and a toxic form of autophagy which through mitochondrial dysfunction with the release of both cytochrome c and AIF and a necroptotic form of cell death [3].

Follow-on studies then linked the effects of AR-12 on tumor cell biology to regulation of the chaperone proteins HSP90, GRP78 and HSP70 [4]. It was observed that AR-12 reduced the protein levels of HSP90 and GRP78 but stimulated HSP70 expression in a PERK-dependent fashion, as measured using the standard techniques of SDS PAGE and western immuno-blotting. Others independently confirmed our findings regarding AR-12 and the induction of cytotoxic ER stress [5]. Finally, as AR-12 down-regulates the PERK inhibitory chaperone GRP78, and as the induction of toxic autophagy was PERK dependent, we investigated the role of reduced GRP78 expression caused by AR-12 in the regulation of drug toxicity. AR-12 did not alter the transcription of GRP78 to any significant extent but instead destabilized the GRP78 protein itself, considerably reducing its half-life as assessed by western blotting from > 24 hours to approximately 10 hours [6]. Over-expression of GRP78 prevented AR-12 induced PERK activation; autophagy induction, and tumor cell killing.

Our studies published in 2014 and 2015 using AR-12 have further emphasized the importance of chaperones and particularly GRP78 in the cell biology effects of OSU-03012 (AR-12). We discovered that phosphodiesterase 5 inhibitors such as sildenafil (Viagra) and tadalafil (Cialis) synergized with OSU-03012 to kill a wide variety of tumor cells through enhanced PERK-dependent ER stress and autophagy, as well as through activation of the death receptor CD95 (FAS-R) [7]. Similar data were also obtained with the parent drug of OSU-03012, celecoxib, and also with the multi-kinase inhibitors sorafenib, regorafenib and pazopanib [8, 9]. Our *in vitro* and animal based studies are now two open clinical trials; in all solid tumor patients (NCT02466802) where patients are receiving increasing once daily (QD) dosing with regorafenib and sildenafil; in recurrent glioblastoma patients (NCT01817751) where they receive sorafenib, sildenafil and valproate twice daily (BID).

Multiple chaperone proteins play essential roles in maintaining protein stability and cell signaling, most particularly in tumor cells which generally express much greater amounts of cellular protein than non-transformed cells. e.g. multiple myeloma cells. Thus some chaperone proteins, e.g. HSP90, have been the target for many developmental therapeutic chemists and also tumor cell biology researchers. In the field of virology, chaperone proteins, particularly GRP78 have also been recognized as playing essential roles in the life cycles of both DNA and RNA viruses [10, 11]. Based on the fact our cancer biology data with chaperones and OSU-03012 was congruent with the wider literature exploring the roles of chaperones in virus biology, we recently performed studies to determine whether OSU-03012 could alter virus reproduction *in vitro*. Using OSU-03012 or using the multi-kinase inhibitors sorafenib (Nexavar) and pazopanib (Votrient) we determined that the expression of multiple chaperones was rapidly reduced following drug treatment; effects that were magnified by sildenafil [12, 13]. Using molecular tools we proved that the down-regulation of GRP78 was an essential property of these drugs in preventing virus reproduction.

The present studies were initiated to determine whether OSU-03012 or sorafenib or pazopanib altered the expression/localization of additional chaperone proteins and to identify novel targets of OSU-03012 using a proteomics approach. Novel targets from the mass spectrometry studies were then tested as OSU-03012 targets *in vitro* and whether such targets regulated tumor cell biology.

## RESULTS

**Figure 1:**
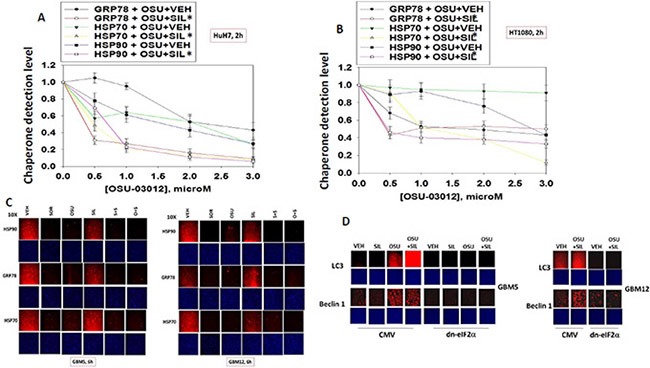
Assessing chaperone expression by immuno-fluorescence and SDS PAGE / western blotting generates divergent data after OSU-03012 or sorafenib treatment, Part 1 (**A** and **B**) HuH7 cells and HT1080 were treated with vehicle, OSU-03012 (0–3.0 μM) and/or sildenafil (2 μM) for 2 h after which cells were fixed in place and permeabilized using 0.5% Triton X100. Immuno-fluorescence was performed to detect the expression levels of GRP78, HSP70 and HSP90. The relative fluorescence intensity value from 40 different cells from each condition was determined using Hermes system software (+/− SEM). (**C**) GBM5 and GBM12 cells were treated with vehicle, OSU-03012 (2.0 μM) / sorafenib (2 μM) and/or sildenafil (2 μM) for 2 h after which cells were fixed in place and permeabilized using 0.5% Triton X100. Immuno-fluorescence was performed to detect the expression levels of GRP78, HSP70 and HSP90. (**D**) As indicated, GBM5, GBM6 and GBM12 cells were transfected with either an empty vector plasmid (CMV) or a plasmid to express dominant negative eIF2α S51A. Twenty four h after transfection cells were treated with vehicle, OSU-03012 (2.0 μM) and/or sildenafil (2 μM) for 6 h after which cells were fixed in place and permeabilized using 0.5% Triton X100. Immuno-fluorescence was performed to detect the expression levels of LC3 (ATG8) and Beclin1 (ATG6).

**Figure 2:**
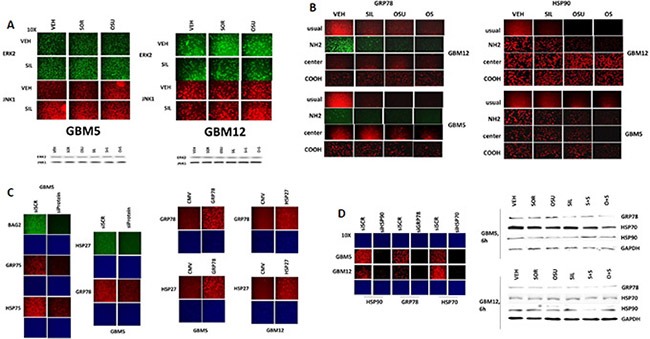
Assessing chaperone expression by immuno-fluorescence and SDS PAGE/western blotting generates divergent data after OSU-03012 or sorafenib treatment, Part 2 (**A**) GBM5 and GBM12 cells were treated with vehicle, OSU-03012 (2.0 μM) and/or sildenafil (2 μM) for 6 h after which: (a) in 96 well plates, cells were fixed in place and permeabilized using 0.5% Triton X100. Immuno-fluorescence was performed to detect the expression levels of ERK2 and JNK1; (b) cells were lysed with bromophenol blue buffer and subjected to SDS PAGE followed by immuno-blotting to detect the expression levels of ERK2 and JNK1. (**B**) GBM12 cells were treated with vehicle, OSU-03012 (2.0 μM) / sorafenib (2 μM) and/or sildenafil (2 μM) for 2 h after which cells were fixed in place and permeabilized using 0.5% Triton X100. Immuno-fluorescence was performed to detect the expression levels of GRP78 and HSP90, using antibodies that recognize epitopes in the NH_2_-terminus; COOH-terminus; and in the middle of the proteins, as well as our previously utilized “*usual*” antibody. (**C**) **Left:** GBM5 cells were transfected with a scrambled siRNA molecule or siRNA molecules to knock down the expression of BAG2, GRP75, HSP75, HSP27 or GRP78. Twenty four h after transfection cells were fixed in place and permeabilized using 0.5% Triton X100. Immuno-fluorescence was performed to detect the expression levels of BAG2, GRP75, HSP75, HSP27 or GRP78, as indicated. **Right:** GBM5 and GBM12 cells were transfected with an empty vector plasmid (CMV) or plasmids to express GRP78 or HSP27. Twenty four h after transfection cells were fixed in place and permeabilized using 0.5% Triton X100. Immuno-fluorescence was performed to detect the expression levels of HSP27 or GRP78, as indicated. (**D**) **Left:** GBM5 and GBM12 cells were transfected with a scrambled siRNA molecule or siRNA molecules to knock down the expression of HSP90, GRP78 or HSP70. Twenty four h after transfection cells were fixed in place and permeabilized using 0.5% Triton X100. Immuno-fluorescence was performed to detect the expression levels of HSP90, HSP70 or GRP78, as indicated. **Right:** GBM5 and GBM12 cells were treated with vehicle, OSU-03012 (2.0 μM) or sorafenib (2 μM) and/or sildenafil (2 μM) for 6 h. Cells were then lysed with bromophenol blue buffer and subjected to SDS PAGE followed by immuno-blotting to detect the expression levels of GRP78, HSP70, HSP90 and GAPDH.

Treatment of HuH7 hepatoma or HT1080 sarcoma cells with OSU-03012 +/− sildenafil or sorafenib +/− sildenafil resulted in a rapid dose- and time-dependent reduction in the detection of the ATP binding and ATPase competent chaperone proteins HSP90, GRP78 and HSP70 in fixed *in situ* cells as judged using immuno-fluorescence with 0.5% (v/v) Triton-X100 for cell permeabilization from a Hermes WiScan wide field microscope (Figure 1A and 1B). Similar changes in the apparent levels of HSP90, GRP78 and HSP70 were also obtained using immuno-fluorescence in the primary human glioblastoma isolates GBM5 (PDGFRα+) and GBM12 (mutant active full length ERBB1) (Figure 1C). Expression of dominant negative eIF2α blocked OSU-03012–induced expression of LC3 and Beclin1 (Figure 1D). We have previously published that OSU-03012 and [OSU-03012 + sildenafil] kills tumor cells through a PERK-dependent form of toxic autophagy [3]. In contrast to the altered detection /expression levels of chaperone proteins in Figure 1A–1D using *in situ* immuno-fluorescence staining, the detection of the kinases ERK2 and JNK1 which are of course also ATP binding proteins with ATPase activity, were not altered by any of drug treatments at this time as judged using either immuno-fluorescence or SDS PAGE followed by traditional western blotting (Figure 2A).

Using antibodies raised against different epitopes within HSP90 and GRP78 we observed that treatment of cells with OSU-03012 reduced the immuno-fluorescence detection of GRP78 and HSP90 with antibodies raised against a synthetic peptide corresponding to residues 270–300 in human GRP78 and against synthetic peptides surrounding E289 of human HSP90; the standard antibodies we have used to detect these proteins in multiple other manuscripts. We also observed reduced immuno-fluorescence using antibodies that were generated to recognize epitopes even nearer the NH_2_-termini of both proteins (Figure 2B). However, using antibodies generated against sequences in the central region, i.e. G584 in GRP78 and between residues 535–732 in HSP90; or in the COOH-termini, i.e. residues 603–617 in GRP78 and near the TPR acceptor site dimerization residues MEEVD in HSP90, modest to no alterations in the immuno-fluorescence signal was observed.

Using siRNA knock-down or protein over-expression we validated that our antibodies to these chaperones were specifically reacting with their targets (Figure 2C and 2D panels to the left). In contrast to our data with the protein kinases ERK2 and JNK1, where data obtained from immuno-fluorescence and SDS PAGE/western blotting were congruent, the detection of HSP90, GRP78 and HSP70 chaperone expression by immuno-fluorescence and SDS PAGE / western blotting using the same antibodies at the 6 h time point were not in agreement (Figure 2D panels to the right; *cf* Figure 1A–1D).

Previously, using SDS PAGE / western blotting we had demonstrated that OSU-03012 destabilizes GRP78 protein resulting in a half-life reduction from > 24 h to ∼10 h despite modestly increasing GRP78 promoter activity [6]. Thus to the naked eye a substantial decline in GRP78 levels, as assessed by western blotting, only became evident 6-12 h after OSU-03012 exposure. This contrasts with the present immuno-fluorescence and western blot data in Figures 1A–1D and 2A–2D where OSU-03012 as a single agent dramatically reduces the immuno-fluorescence detection of HSP90, GRP78 and HSP70 within 6 h but does not alter the signal detected by western blotting. As some types of cellular membrane are not permeabilized by Triton X-100 we examined immuno-fluorescence staining in fixed cells after they had been treated with β-cyclodextrin or with CHAPS. Treatment of cells with drugs followed by fixing and membrane permeabilization with 10 mM β-cyclodextrin or 1% (w/v) CHAPS did not alter the apparent observed decline in HSP90, GRP78, HSP70 and HSP27 levels as determined by immuno-fluorescence (data not shown). This data, together with our detection data using antibodies raised against different epitopes in the chaperone proteins suggests that the tertiary structure of chaperone proteins may be rapidly altered by OSU-03012. Moreover, these data indicate that standard western blotting techniques involving denaturation are not optimal for detailed studies of how chaperone structure may vary in cells upon drug exposure.

**Figure 3:**
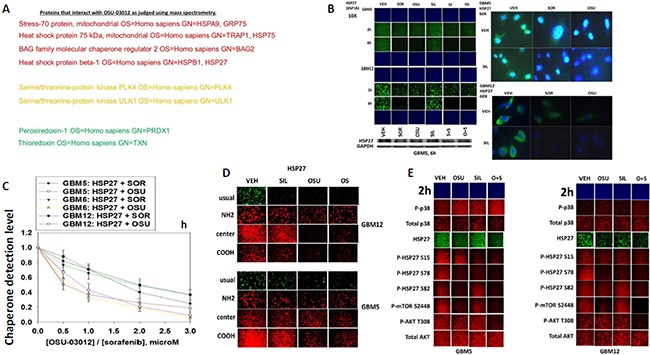
Proteomic and cell biology analyses of AR-12 interacting proteins (**A**) As described in the Methods, AR-12 conjugated via biotin to sepharose beads was used to capture proteins from a whole cell lysate and those proteins specifically associating with beads in an AR-12–dependent fashion were determined after proteolytic digestion in a mass spectrometer. (**B**) GBM5 and GBM12 cells were treated with vehicle, OSU-03012 (2.0 μM) / sorafenib (2.0 μM) and/or sildenafil (2 μM) for 6 h after which: (a) in 96 well plates, cells were fixed in place and permeabilized using 0.5% Triton X100. Immuno-fluorescence was performed to detect the expression level of HSP27 presented at 10X and 60X magnification; (b) cells were lysed with bromophenol blue buffer and subjected to SDS PAGE followed by immuno-blotting to detect the expression level of HSP27. (**C**) GBM5, GBM6 and GBM12 cells were treated with vehicle, OSU-03012 (0–3.0 μM) or sorafenib (0–3.0 μM) for 3 h after which cells were fixed in place and permeabilized using 0.5% Triton X100. Immuno-fluorescence was performed to detect the expression level of HSP27. The relative fluorescence intensity value from 40 different cells from each condition was determined using Hermes system software (+/− SEM). (**D**) GBM12 cells were treated with vehicle, OSU-03012 (2.0 μM) / sorafenib (2 μM) and/or sildenafil (2 μM) for 2 h after which cells were fixed in place and permeabilized using 0.5% Triton X100. Immuno-fluorescence was performed to detect the expression level of HSP27, using antibodies that recognize epitopes in the NH_2_-terminus; COOH-terminus; and in the middle of the protein, as well as our previously used “usual” antibody. (**E**) GBM5 and GBM12 cells were treated with vehicle, OSU-03012 (2.0 μM) and/or sildenafil (2 μM) for 6 h after which cells were fixed in place and permeabilized using 0.5% Triton X100. Immuno-fluorescence was performed to detect the total expression level of AKT and p38 MAPK, and the phosphorylation levels of AKT T308; p38 MAPK; mTOR S2448; HSP27 S15; HSP27 S78; and HSP27 S82.

We next sought to determine whether other cellular proteins, including those in the wider chaperone super-family, interacted with OSU-03012. As a proteomics collaborator was not available at Massey Cancer Center we collaborated with colleagues at the NIH. At least four chaperone family proteins were co-precipitated by OSU-03012 (Figure 3A). Two protein kinases and two proteins that regulate cellular redox status were also identified. Based on these discoveries we performed additional immuno-fluorescence studies to determine the impact of OSU-03012 and of sorafenib on the expression and/or phosphorylation and localization of these proteins. The expression of the small chaperone HSP27, which lacks an ATP binding site, was reduced by both OSU-03012 and by sorafenib, with our immuno-fluorescence data in general agreement with data generated via standard western blotting (Figure 3B). Based on 60X magnification images of the cells, HSP27 appeared to be both widely distributed in the cytosol and also in globular particulate formations in vehicle treated cells, whereas drug treatments abolished any particulate chaperone staining. In drug-dose-response studies OSU-03012 at a concentration of 1 μM was able to reduce HSP27 detection levels in three glioblastoma cell isolates within 3 h (Figure 3C). Using antibodies raised against different epitopes within HSP27 we observed that treatment of cells with OSU-03012 reduced the immuno-fluorescence detection of HSP27 with an antibody that was raised against full-length HSP27 protein, i.e. our “*usual*” antibody; no precise epitope provided by the supplier, and antibodies that recognize the central region of the protein, i.e. amino acids 32–108, and a synthetic peptide raised against the COOH-terminus of the protein (Figure 3D). However, using an antibody specifically generated against the NH_2_-terminus of the HSP27 protein, residues 2–12, no apparent drug-induced alteration in the immuno-fluorescence signal was observed.

OSU-03012 and [OSU-03012 + sildenafil] treatment very modestly enhanced the stoichiometry of p38 MAPK phosphorylation (Figure 3E). OSU-03012 as a single agent increased the stoichiometry of HSP27 phosphorylation at Serine 15, but not at Serine 78 or at Serine 82; i.e. total expression of HSP27 declined whereas HSP27 phosphorylation did not alter, hence the stoichiometry of HSP27 phosphorylation rose. Our data also argues that Serine 78 phosphorylation declined after sildenafil exposure. Of particular note were the side-by-side comparisons of AKT Threonine 308 phosphorylation and mTOR Serine 2448 phosphorylation. As we have previously shown for AKT Threonine 308 phosphorylation in these glioblastoma cells, OSU-03012 as a single agent had little to no obvious effect on T308 phosphorylation levels (Figure 3E). In contrast, OSU-03012 as a single agent strongly reduced mTOR Serine 2448 phosphorylation. Thus our data argues that OSU-03012 causes a greater degree of mTOR dephosphorylation when compared to its more modest effect on AKT dephosphorylation, implying that the regulation of mTOR activity by the drug is likely to be more biologically important than that of AKT.

**Figure 4:**
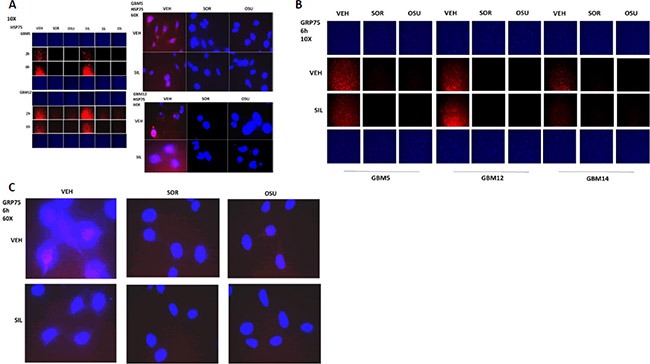
HSP75 and GRP75 are AR-12 interacting proteins (**A**) GBM5 and GBM12 cells were treated with vehicle, OSU-03012 (2.0 μM)/sorafenib (2.0 μM) and/or sildenafil (2 μM) for 6 h after which cells were fixed in place and permeabilized using 0.5% Triton X100. Immuno-fluorescence was performed to detect the expression level of HSP75 presented at 10X and 60X magnification. (**B** and **C**) GBM5 and GBM12 cells were treated with vehicle, OSU-03012 (2.0 μM)/sorafenib (2.0 μM) and/or sildenafil (2 μM) for 6 h after which cells were fixed in place and permeabilized using 0.5% Triton X100. Immuno-fluorescence was performed to detect the expression level of GRP75 presented at 10X and 60X magnification.

We next examined the other two chaperone proteins shown to interact with OSU-03012 in Figure 3A; HSP75 and GRP75 (Figure 4A and 4B). The expression of both HSP75 and GRP75 was reduced by treatment with OSU-03012 or with sorafenib. Based on 60X magnification images of the cells, HSP75 appeared to be both widely distributed in the cytosol and also to have peri-nuclear staining in vehicle treated cells, whereas drug treatments abolished any peri-nuclear staining (Figure 4A and 4C).

**Figure 5:**
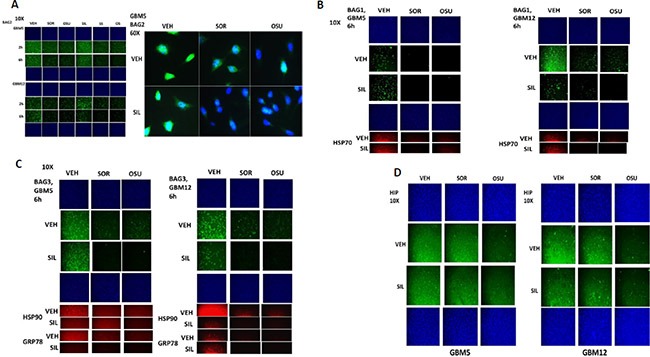
BAG2 is an AR-12 interacting protein (**A**) GBM5 and GBM12 cells were treated with vehicle, OSU-03012 (2.0 μM)/sorafenib (2.0 μM) and/or sildenafil (2 μM) for 6 h after which cells were fixed in place and permeabilized using 0.5% Triton X100. Immuno-fluorescence was performed to detect the expression level of BAG2 presented at 10X and 60X magnification. (**B** and **C**) GBM5 and GBM12 cells were treated with vehicle, OSU-03012 (2.0 μM)/sorafenib (2.0 μM) and/or sildenafil (2 μM) for 6 h after which cells were fixed in place and permeabilized using 0.5% Triton X100. Immuno-fluorescence was performed to detect the expression level of BAG1 and BAG3 presented at 10X magnification. (**D**) GBM5 and GBM12 cells were treated with vehicle, OSU-03012 (2.0 μM)/sorafenib (2.0 μM) and/or sildenafil (2 μM) for 6 h after which cells were fixed in place and permeabilized using 0.5% Triton X100. Immuno-fluorescence was performed to detect the expression level of HIP presented at 10X magnification.

BCL-2 associated athanogene (BAG) proteins are chaperone regulator/adaptor molecules that compete with the protein HIP for binding to the ATPase domain of HSP70 family chaperones, including HSP70, and regulate the trafficking of misfolded proteins to the proteasome or to autophagy. In our proteomic studies the protein BAG2 associated with OSU-03012, and both OSU-03012 and sorafenib reduced BAG2 protein expression (Figure 5A). In addition, treatment of cells with OSU-03012 or sorafenib altered the granular/punctate staining of BAG2 with its localization becoming more uniform. There are two other members of the BAG family, BAG1 and BAG3. Treatment of cells with OSU-03012 or sorafenib also reduced BAG1 and BAG3 protein expression (Figure 5B and 5C). The protein HSC70 interacting protein (HIP), together with BAG proteins regulates the ATPase activity and chaperone function of HSP70 family chaperones. OSU-03012, but not sorafenib, reduced HIP expression (Figure 5D).

**Figure 6:**
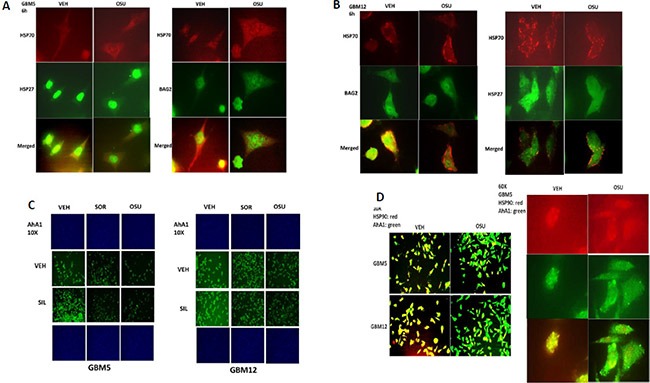
AR-12 disrupts the co-localization of chaperones with their regulatory proteins (**A** and **B**) GBM5 and GBM12 cells were treated with vehicle control or with OSU-03012 (2 μM) for 6 h after which cells were fixed in place and permeabilized using 0.5% Triton X100. Immuno-fluorescence was performed to detect the co-localization of HSP70 and BAG2; and HSP70 and HSP27; presented at 60X magnification. (**C**) GBM5 and GBM12 cells were treated with vehicle, OSU-03012 (2.0 μM) / sorafenib (2.0 μM) and/or sildenafil (2 μM) for 6 h after which cells were fixed in place and permeabilized using 0.5% Triton X100. Immuno-fluorescence was performed to detect the expression level of AhA1 presented at 10X magnification. (**D**) GBM12 cells were treated with vehicle control or with OSU-03012 (2 μM) for 6 h after which cells were fixed in place and permeabilized using 0.5% Triton X100. Immuno-fluorescence was performed to detect the co-localization of HSP90 and AhA1 presented at 10X and 60X magnification.

In protein-protein co-localization studies, where red staining overlapping green staining co-localizes to generate a yellow fluorescent signal, we noted that OSU-03012 reduced the co-localization of BAG2 and HSP70 and of HSP27 and HSP70 in GBM cells (reduced yellow staining; increased individual green and red staining) (Figure 6A and 6B). In a manner similar to the BAG proteins and HIP for HSP70; the protein AhA1 regulates the ATP loading of HSP90. Treatment of cells with OSU-03012/sorafenib with or without sildenafil modestly reduced the detection of AhA1 by immuno-fluorescence (Figure 6C). In co-localization studies we noted that OSU-03012 treatment reduced the co-localization of AhA1 and HSP90 in GBM cells (reduced yellow staining; increased green and red staining), with the AhA1 and HSP90 association in punctate bodies decreased by drug exposure (Figure 6D).

**Figure 7:**
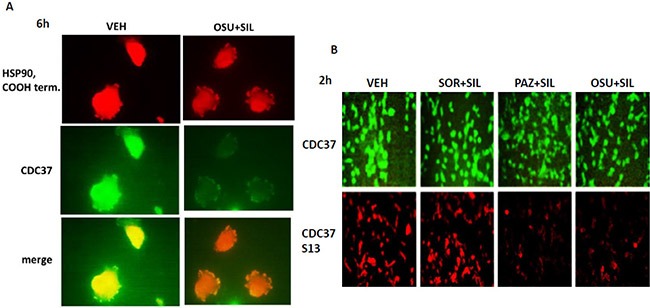
Drug combination treatments reduce CDC37 Serine 13 phosphorylation (**A**) GBM12 cells were treated for 6 h with OSU-03012 (2.0 μM) and sildenafil (2.0 μM) for 6 h after which cells were fixed in place and permeabilized using 0.5% Triton X100. Immuno-fluorescence was performed to detect the expression levels of CDC37 and HSP90 and the co-localization of CDC37 and HSP90 at 60X magnification. (**B**) GBM12 cells were treated with vehicle, OSU-03012 (2.0 μM) / sorafenib (2.0 μM) / pazopanib (2.0 μM) and/or sildenafil (2 μM) for 6 h after which cells were fixed in place and permeabilized using 0.5% Triton X100. Immuno-fluorescence was performed to detect the expression level of CDC37 and the Serine 13 phosphorylation level in CDC37 presented at 10X magnification.

HSP90 function, in addition to being regulated by AhA1, is also controlled by the essential co-chaperone CDC37. CDC37 facilitates HSP90 chaperoning of cyclin dependent kinases, IκB kinases and eIF2α kinases, and is itself phosphorylated on Serine 13 by casein kinase 2 [23, 24]. Serine 13 phosphorylation stabilizes CDC37 into a more compact conformation with enhanced secondary structure and in yeast models mutation of this site causes severe growth deficiency and reduced expression of multiple CDC37 client proteins. In co-localization studies we noted that OSU-03012 after a 6 h treatment reduced the total expression and the co-localization of CDC37 with HSP90 in GBM cells (reduced yellow staining; increased green and red staining) (Figure 7A). Treatment of cells with [OSU-03012 + sildenafil] or [pazopanib + sildenafil] for 2 h did not alter total CDC37 expression but decreased CDC37 Serine 13 phosphorylation levels (Figure 7B).

**Figure 8:**
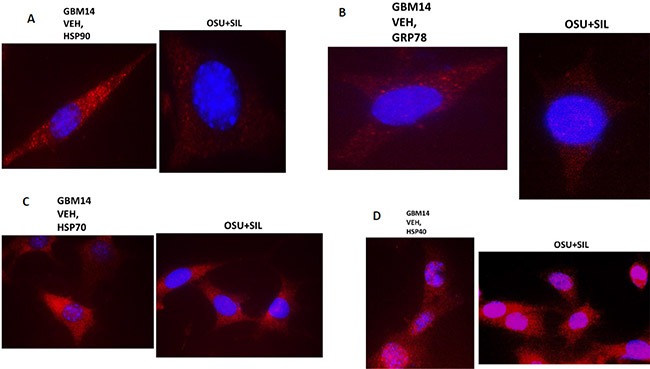
The sub-cellular distribution and morphology of chaperone complexes in cells before and after treatment with [OSU-03012 + sildenafil], Part 1 (**A**–**D**) GBM14 cells were treated with either vehicle control or OSU-03012 (2 μM) and sildenafil (2 μM) for 6 h. Cells were fixed in place and permeabilized using 0.5% Triton X100. Immuno-fluorescence was performed to detect the expression levels of HSP90; GRP78; HSP70; and HSP40, presented at 60X magnification.

We then performed additional analyses on other chaperone proteins, also at 60X magnification, to determine whether the localization of the chaperone in the cell was being altered following [OSU-03012 + sildenafil] treatment. In vehicle treated glioma cells HSP90 and GRP78 were localized in multiple small punctate bodies, whose staining was markedly reduced 6 h after [OSU-03012 + sildenafil] treatment (Figure 8A and 8B). In contrast, HSP70 and HSP40 localization was not altered by drug exposure (Figure 8C and 8D).

**Figure 9:**
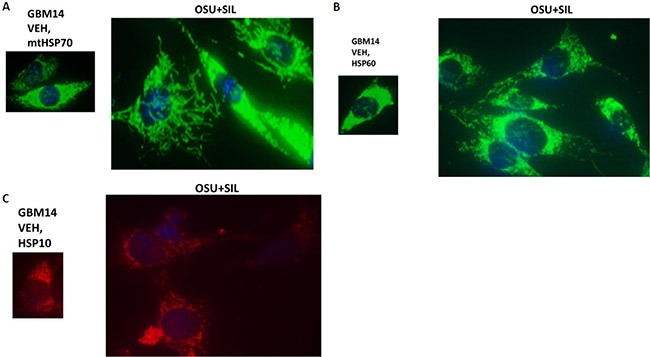
The sub-cellular distribution and morphology of chaperone complexes in cells before and after treatment with [OSU-03012 + sildenafil], Part 2 (**A–C**) GBM14 cells were treated with either vehicle control or OSU-03012 (2 μM) and sildenafil (2 μM) for 6 h. Cells were fixed in place and permeabilized using 0.5% Triton X100. Immuno-fluorescence was performed to detect the expression levels of mitochondrial HSP70; HSP60; and HSP10, presented at 60X magnification.

Mitochondrial HSP70 (mtHSP70) staining in vehicle control cells exhibited a dense mackerel staining pattern as would be expected for mitochondrial localization which was dramatically altered after [OSU-03012 + sildenafil] exposure such that mitochondrial HSP70 staining occurred in very long vermiform projections (Figure 9A). In a manner reminiscent of the staining for mitochondrial HSP70, HSP60 and HSP10 protein staining also became more diffuse and vermiform after [OSU-03012 + sildenafil] exposure (Figure 9B and 9C). Very similar chaperone immuno-fluorescence data have been recently previously published by the Dent laboratory when treating tumor cells with [sorafenib + sildenafil] [22].

**Figure 10:**
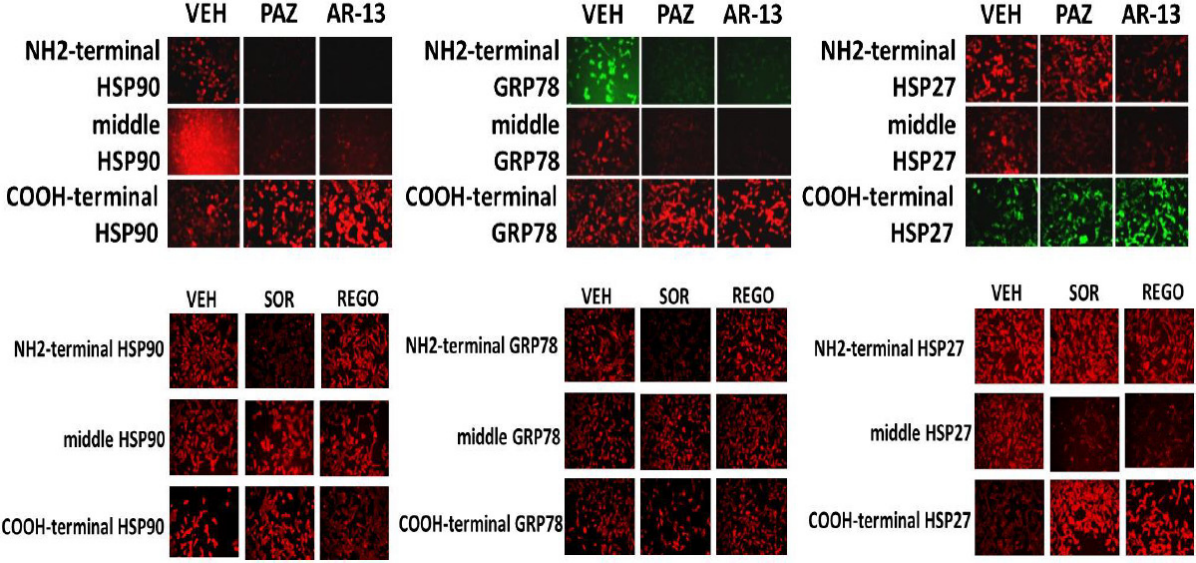
Chaperone conformation is regulated by OSU-03012 GBM12 cells were treated with vehicle or OSU-03012 (2 μM); sorafenib (2 μM); regorafenib (2 μM); pazopanib (2 μM); AR-13 (2 μM); celecoxib (2 μM) for 2 h. Cells were fixed in place and permeabilized using 0.5% Triton X100. Immuno-fluorescence was performed to detect the expression levels of HSP90, GRP78 and HSP27, using antibodies that recognize epitopes in the NH_2_-terminus; COOH-terminus; and in the middle of the proteins.

**Figure 11:**
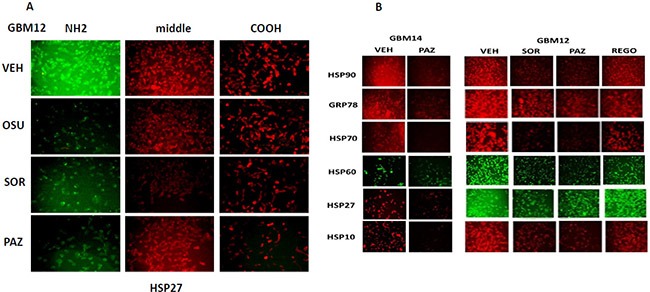
Modulation of HSP70 NH_2_-terminal detection by OSU-03012 and other drugs (**A**) GBM12 cells were treated with vehicle or sorafenib (2 μM); regorafenib (2 μM); OSU-03012 (2 μM); AR-13 (2 μM); pazopanib (2 μM) for 2 h. Cells were fixed in place and permeabilized using 0.5% Triton X100. Immuno-fluorescence was performed to detect the expression levels of HSP70 using antibodies raised to detect the NH_2_-terminus, the central region, and COOH terminus of the protein. (**B**) GBM14 and GBM12 cells were treated with vehicle or sorafenib (2 μM); regorafenib (2 μM); pazopanib (2 μM) for 2 h. Cells were fixed in place and permeabilized using 0.5% Triton X100. Immuno-fluorescence was performed to detect the expression levels of the indicated chaperone proteins.

**Figure 12:**
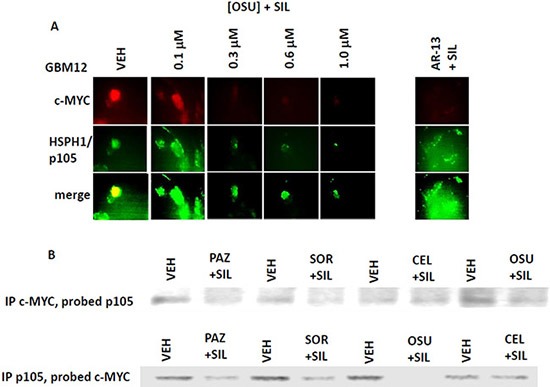
Co-localization of the NH_2_-terminal and COOH-terminal directed HSP70 antibodies (**A**) GBM12 cells were treated with vehicle or [OSU-03012 (0-1.0 μM) + sildenafil (2.0 μM)] or with [AR-13 (1 μM) + sildenafil (2 μM)] for 6 h. Cells were then fixed in place and permeabilized using 0.5% Triton X100. Immuno-fluorescence was performed to detect the total expression and the co-localization of c-MYC and HSPH1/p105. (**B**) GBM12 cells were treated with vehicle or [OSU-03012 (2.0 μM) + sildenafil (2.0 μM)] for 30 min. Cells were lysed and portions subjected to immuno-precipitation for c-MYC or for HSPH1/p105, followed by SDS PAGE and western blotting to determine the co-precipitation of HSPH1/p105 with c-MYC and of c-MYC with HSPH1/p105.

We next determined whether sorafenib, pazopanib and the sulfonamide analogue of OSU-03012 (AR-12), called AR-13, also altered our ability to detect HSP90, GRP78 and HSP27 by *in situ* immuno-fluorescence. As was previously observed for OSU-03012, sorafenib/pazopanib/AR-13 reduced the immuno-fluorescence detection of HSP90, HSP70 and GSP78 using an NH_2_-terminal specific antibody but not using antibodies directed against epitopes in the middle and COOH-terminal portions of the proteins (Figure 10). As a single agent, regorafenib was ineffective at changing the immuno-fluorescence detection of the NH_2_-termini of HSP90, HSP70 or GRP78 (Figures 10 and 11A). Upon examination of other chaperone proteins we also observed that sorafenib and pazopanib could reduce the detection of native proteins by immuno-fluorescence, an effect largely not present in cells treated with regorafenib (Figure 11B). In a dose-dependent fashion [OSU-03012 + sildenafil] reduced the expression of HSPH1/p105 and a protein it chaperones, c-MYC (Figure 12A). AR-13 and sildenafil also reduced the total expression and co-localization of p105 and c-MYC. In immuno-precipitates, [OSU-03012 + sildenafil] reduced the co-precipitation of c-MYC with p105 and of p105 with c-MYC (Figure 12B).

**Figure 13:**
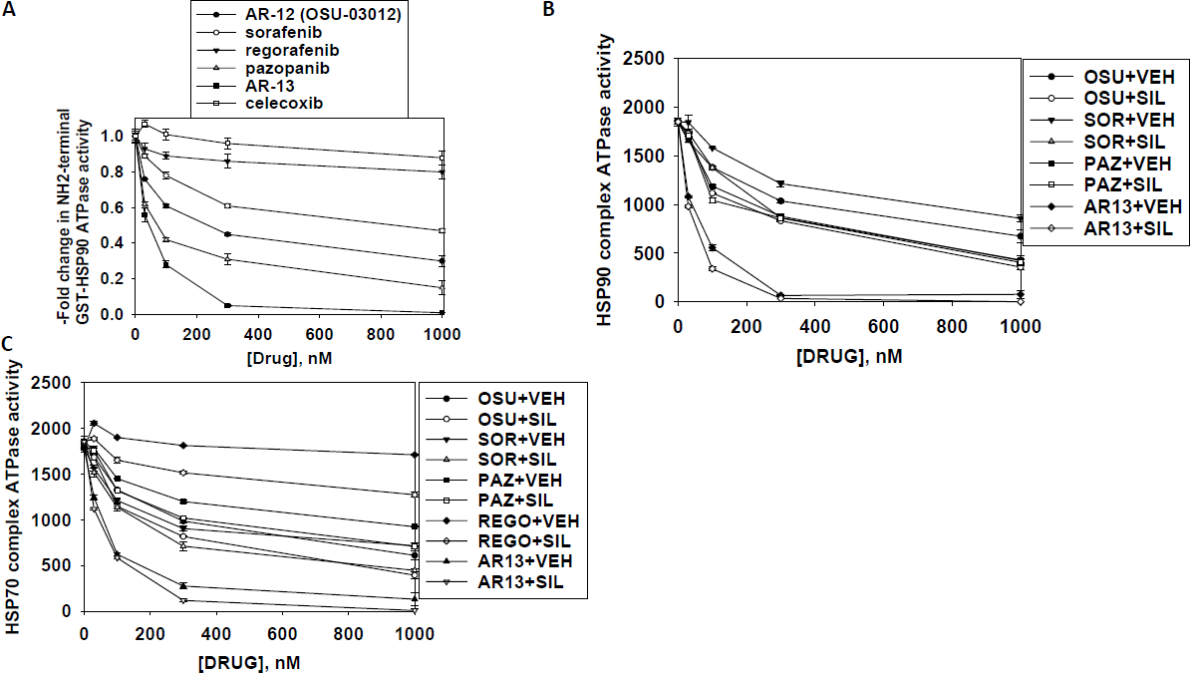
Sildenafil enhances the ATPase inhibitory effects of regorafenib, sorafenib, pazopanib, AR-13 and OSU-03012 (**A**) A GST-HSP90 NH_2_-terminal fragment containing the ATP binding domain of the chaperone was synthesized in *E. coli* and purified from other bacterial proteins using glutathione sepharose. The GST-HSP90 NH_2_-terminal fragment protein *was not* eluted off the sepharose beads. Equal portions of beads were immediately aliquoted into individual wells in a 96 well plate. Beads were resuspended in kinase reaction buffer containing vehicle control; OSU-03012; sorafenib tosylate; regorafenib; pazopanib; AR-13; celecoxib (30 nM; 100 nM; 300 nM; 1 μM) in triplicate, and incubated for 30 min at 37°C. The reaction was started by addition of ATP-lite substrate. The plate was removed from the incubator and placed into a Vector 3 plate reader to determine the luminescence of the reactions under each treatment condition (*n* = 3 (× 3) +/− SEM). (**B** and **C**) GBM12 cells were transfected with a plasmid to express HSP70-GFP or to express FLAG-tagged HSP90. Twenty four h after transfection cells were treated with vehicle control or sildenafil (2 μM) for 1 h. Chaperone proteins were immuno-precipitated using their tags in the presence of phosphatase inhibitors. Equal portions of precipitate sepharose beads were immediately aliquoted into individual wells in a 96 well plate. Beads were resuspended in ATPase reaction buffer containing vehicle control; OSU-03012; sorafenib tosylate; regorafenib; pazopanib; AR-13; (30 nM; 100 nM; 300 nM; 1 μM) in triplicate, and incubated for 30 min at 37°C. The reaction was started by addition of ATP-lite substrate. The plate was removed from the incubator and placed into a Vector 3 plate reader to determine the luminescence of the reactions under each treatment condition (*n* = 3 (× 3) +/− SEM).

As heat shock proteins co-precipitated with OSU-03012 in our proteomic studies, we investigated whether OSU-03012 altered chaperone ATPase activity. We transformed bacteria with a plasmid to make a GST fusion protein of the NH_2_-terminal portion of HSP90; the domain that contains the ATP binding site and ATPase activity of the chaperone. Equal portions of the NH_2_-terminal portion of HSP90 were subjected to ATPase activity analyses. OSU-03012 reduced chaperone ATPase activity, as measured on the isolated purified NH_2_-terminal HSP90-GST protein fragment *in vitro* with an IC_50_ of ∼200 nM in the presence of 0.1 mM ATP substrate (Figure 13A). Similar data were obtained with the multi-kinase inhibitor sorafenib and with the multi-kinase inhibitor pazopanib, but not the fluorinated analogue of sorafenib, regorafenib, or the parent compound of OSU-03012, celecoxib. Remarkably, AR-13 inhibited HSP90 chaperone ATPase activity, with an IC_50_ of ∼40 nM. We then expressed tagged forms of HSP90 and HSP70 in eukaryotic cells, with cells treated for 1 h with either vehicle or sildenafil. Equal portions of HSP90 and HSP70 were subjected to ATPase activity analyses. We found that equal quantities HSP90 and HSP70 proteins isolated using their engineered tags from sildenafil treated cells unexpectedly had near identical ATPase activities than those from vehicle control treated cells (Figure 13B and 13C). As was previously observed for bacterial expressed HSP90 ATPase activity; the ATPase activities of eukaryotic synthesized HSP90 and HSP70 were also inhibited by OSU-03012, sorafenib and pazopanib; but were not inhibited by regorafenib. For OSU-03012, sildenafil enhanced its inhibitory effects against both the HSP90 and HSP70 ATPase activities. For sorafenib, sildenafil enhanced its inhibitory effects against both the HSP90 and HSP70 ATPase activities. For pazopanib, sildenafil did not further enhance its potent inhibition of the HSP90 ATPase but did enhance its inhibitory effect against the HSP70 ATPase. Pazopanib was a more potent inhibitor of the HSP90 ATPase activity than sorafenib whereas sorafenib was a more potent inhibitor of the HSP70 ATPase activity than pazopanib. For regorafenib, sildenafil did not enhance its HSP90 ATPase inhibitory activity but did enhance its inhibitory properties against the HSP70 ATPase. Perhaps most interestingly, the novel analogue AR-13 inhibited the ATPase activities associated with the HSP90 and the HSP70 chaperone complexes more efficaciously than it did the bacterial synthesized NH_2_-terminal portion of HSP90.

**Figure 14:**
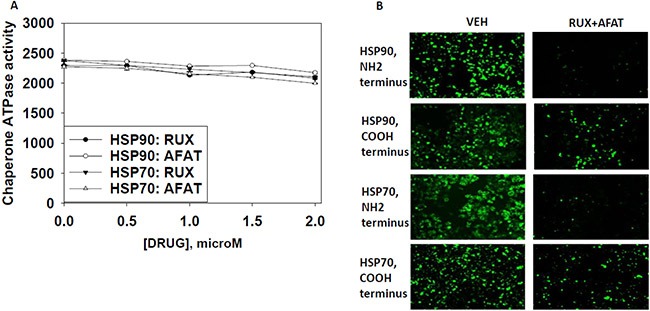
Afatinib nor ruxolitinib have ATPase inhibitory effects against HSP90 or HSP70 (**A**) GBM12 cells were transfected with a plasmid to express HSP70-GFP or to express FLAG-tagged HSP90. Twenty four h after transfection chaperone proteins were immuno-precipitated using their tags. Equal portions of precipitate sepharose beads were immediately aliquoted into individual wells in a 96 well plate. Beads were resuspended in ATPase reaction buffer containing vehicle control; ruxolitinib; or afatinib (500 nM; 1000 nM; 1500 nM; 2 μM) in triplicate, and incubated for 30 min at 37°C. The reaction was started by addition of ATP-lite substrate. The plate was removed from the incubator and placed into a Vector 3 plate reader to determine the luminescence of the reactions under each treatment condition (*n* = 3 (× 3) +/− SEM). (**B**) GBM12 cells were treated with vehicle or [afatinib (1 μM) and ruxolitinib (1 μM)] for 6 h. Cells were fixed in place and permeabilized using 0.5% Triton X100. Immuno-fluorescence was performed to detect the expression levels of HSP70 and of HSP90 using antibodies raised to detect the NH_2_-termini and COOH termini of the proteins.

Based on the fact that sorafenib and pazopanib are well-known to be multi-S/T/Y kinase inhibitors we wished to prove or refute whether other tyrosine kinase inhibitors of disparate structures could also inhibit chaperone ATPase activities. Neither the second generation ERBB1/2/4 suicide inhibitor afatinib nor the Janus kinase 1 and 2 inhibitor ruxolitinib could inhibit the ATPase activities associated with HSP90 or HSP70 (Figure 14A). Nevertheless, treatment of cells with [ruxolitinib + afatinib] combined for 6 h did reduce the detection by *in situ* immuno-fluorescence of the NH_2_-termini of HSP90 and HSP70 (Figure 14B). These findings argue that ruxolitinib and afatinib may (somehow) interact with HSP90 and HSP70 so as to alter the tertiary conformation of their NH_2_-termini without impacting on chaperone ATPase function. i.e. conformation change and ATPase inhibition effects may be separate events.

In parallel sets of immuno-precipitates from cells expressing only endogenous levels of HSP90, GRP78 and HSP70 we determined whether OSU-03012, sorafenib, pazopanib or AR-13 could alter the composition of co-precipitating chaperone and other associated proteins. The amount of HSP90, HSP27 and BAG2 in HSP70 immuno-precipitates was reduced by OSU-03012 (Figure S1A). The amount of PERK and WASF3 in GRP78 immuno-precipitates was reduced by OSU-03012. The amount of AhA1 in HSP90 immuno-precipitates was reduced by OSU-03012. Of particular note, less HSP70 co-precipitated with HSP90 in the presence of OSU-03012 that was also associated with reduced HSP70 mobility on SDS PAGE, an effect usually indicative of increased protein phosphorylation. Similar effects were observed when HSP27 was co-precipitated with HSP90, with HSP27 showing reduced SDS PAGE mobility in cells treated with OSU-03012 again suggestive of increased phosphorylation. In cells treated with pazopanib or with AR-13 immuno-precipitation of HSP90, GRP78, HSP70 or HSP27 revealed that the levels of co-precipitating HSP90, GRP78, HSP70 or HSP27 was considerably reduced by each drug (Figure S1B and S1C).

We next determined the impact of OSU-03012 on the elution profile from a gel filtration column of native chaperone protein complex. As a traditional protein biochemist was not available at Massey Cancer Center, we collaborated with a colleague in Australia. HeLa cells were pre-treated for 20 min with OSU-03012 then lysed, and clarified lysates subjected to gel filtration in the presence of vehicle control or OSU-03012. Treatment of cell lysates with OSU-03012 resulted in the chaperone protein HSP27 eluting in lower molecular weight complexes compared to the chaperone elution in vehicle control treated lysates (Figure S2). *n.b.* there also appeared to be less over-all detection of the HSP27 protein itself following gel filtration after OSU-03012 treatment compared to vehicle control, with very similar blotting detection level for the protein loading controls on each piece of nitrocellulose.

As a molecular modeling in silico collaborator was not available at Massey Cancer Center we performed studies with colleagues at Augusta University. In the absence of crystallographic or spectroscopic data, docking, using Autodock Vina, was done to predict the binding mode of OSU-03012 to HSP90 [16–31] (Figure S3A–S3C). Docking of OSU-03012 against an ensemble of structures derived by molecular dynamics (NAMD 2.10) from the high-resolution structure of the NH_2_-terminal domain of human HSP90 in complex with the ATP analog ACP (PDB ref code 3T10) yields two plausible poses. In the first pose, the purine binding pocket is filled by the phenanthroline group of OSU-03012, with the long axis oriented parallel to the surface of the protein. There is also a hydrophobic interaction between the CF_3_ group and the aromatic ring of Tyr139. The protonated amino group in the sidechain interacts with several hydrophilic residues in the α-helix flanking the ATP binding site, either Ser50 or Asp54. In the second pose the long axis of the phenanthroline group is oriented orthogonal to the protein surface. The CF_3_ group makes a hydrophobic contact with the sidechain of Ile110, and the amino group shows similar interactions between Ser50 or Asp54 as in the first pose.

To understand why treatment of cells with OSU-03012 resulted in reduced immuno-detection of the NH_2_-terminal domain of HSP90 we performed accelerated MD of the complex between HSP90 (NH_2_-terminal domain only) and OSU-03012. Starting from the first pose, within a short span of simulated time (30 ns) the phenanthroline group flips to an orthogonal orientation to adopt the second pose. The amino group is seen to interact with several other residues besides Ser50 and Asp54, notably Asp102. A similar simulation was done with apo-HSP90. Comparing between the conformations the protein is seen to adopt with and without ligand, it can be seen that while the conformation of the β-sheets at the back of the ATP binding pocket remains nearly unchanged, the positions of the α-helices are very different. We also determined the distance groups of the OSU-03012 ligand and residues of HSP90 from the MD simulation. In Figure S3D we present data showing the distances between the amino group of OSU-03012 and residues S50, D54 and D102 of HSP90. In Figure S3E we present data showing the distances between the trifluoromethyl group of OSU-03012 and residues I110 and Y138 of HSP90. These structural alterations may account for the differences in chaperone reactivity using antibodies with NH_2_-terminal epitopes.

We next determined whether OSU-03012 could dock into HSP70. The nucleotide-bound structure of HSP70 is tightly closed, but there are structures of HSP70 in complex with nucleotide exchange factors, where the subdomains are enveloping the ADP less tightly. We applied artificial forces to the model to move the atoms to match a target structure, and we did the docking along the way from the starting structure to the target. The most plausible pose is attached; Figure S4A, and is just from the docking. The data presented in Figure S4B has the ATP super-imposed. The phenanthrene group of OSU-03012 is in the nucleotide binding pocked (the arginine residues in this pocket may form cation-pi interactions), the -CF3 group forms hydrophobic interactions with one of the two tyrosine residues nearby, and the amino group in the side-chain interacts with the serine residues that would bind to the phosphate groups of ATP.

We have published several manuscripts demonstrating that low concentrations of OSU-03012 (AR-12) kills tumor cells, and that this effect is enhanced by the PDE5 inhibitor sildenafil. Based on our prior studies in this manuscript, we next determined the relative potency side-by-side of tumor cell killing by OSU-03012 and by AR-13. (Figure S5). AR-13 and OSU-03012 had similar dose-dependent abilities to kill GBM6 and GBM12 cells. However, when combined with sildenafil, AR-13 was significantly more capable of causing tumor cell death than OSU-03012, with concentrations as low as 100 nM causing > 50% tumor cell death. Thus, we next determined whether our drug treatments altered chaperone – chaperone co-localization in cells *in situ* using antibodies for detection whose signal did not show alterations in signal strength after drug exposure. Regorafenib, sorafenib, pazopanib, AR-13 and OSU-03012 reduced the interactions between HSP70 and HSP27; HSP60 and HSP10; and GRP78 and GRP94 (Figure S6). Regorafenib did not alter the co-localization of HSP90 and HSP70. Sorafenib, pazopanib, AR-13 and OSU-03012 all modified the interaction between HSP90 and HSP70 reducing the numbers of punctate sites of co-localization as well as increasing sites of punctate staining that were green or red, not co-localized yellow.

In Figure 3A we demonstrated that two protein kinases associated with OSU-03012; ULK-1 and PLK4. The protein kinase ULK-1 is considered to be an essential gate-keeper kinase for the regulation of autophagosome formation, and whose activity is negatively regulated by Serine 757 phosphorylation by the kinase mTOR [32, 33]. This is of particular interest to us as we know that induction of a toxic form of autophagy plays a central role in OSU-03012 lethality. OSU-03012 or sorafenib reduced the phosphorylation of mTOR at Serine 2448, indicative of mTOR inactivation (Figure S7A). Reduced mTOR S2448 phosphorylation correlated with reduced phosphorylation of ULK-1 at an accepted site of mTOR phosphorylation, Serine 757, as well as appearing to reduce the total expression of ULK-1 protein, which collectively in turn correlated with increased phosphorylation of the autophagosome formation regulatory protein ATG13 at Serine 318 [33–35].

Serine 318 phosphorylation of ATG13 facilitates, together with other protein-protein interaction events, autophagosome formation. When we examined cells at 60X magnification to determine the localization of ULK-1 and ULK-1 S757 phospho-protein we noted a strong peri-nuclear localization and phosphorylation of ULK-1 (Figure S7B). Drug treatments, particularly those containing OSU-03012, demonstrated a marked reduction in the peri-nuclear staining of both ULK-1 and ULK-1 S757. Downstream of ULK-1 we also explored the impact of our drug treatments at 60X on the localization and phosphorylation of ATGATG13 S318. In vehicle or drug treated cells ATG13 protein was detected throughout the cell (Figure S7C). Drug treatments, particularly those containing OSU-03012, increased the levels of peri-nuclear staining ULK-1 S757 concomitant with observation of increased and more intense punctate staining of phospho-ATG13 S318; the punctate staining was reminiscent of autophagosome vesicles detected using expression of an LC3-GFP protein. Transfection of GBM12 cells to express GRP78 both reduced basal phosphorylation levels and prevented the drug-induced phosphorylation of eIF2α and ATG13, but did not prevent drug-induced dephosphorylation of mTOR (Figure S7D). Transfection of GBM12 cells to express HSP27 prevented OSU-03012/sorafenib single agent-induced phosphorylation of eIF2α and ATG13, and prevented the OSU-03012/sorafenib single agent-induced dephosphorylation of mTOR (Figure S7D).

We next performed immuno-fluorescence protein co-localization studies to determine whether phospho-ATG13 S318 or HSP27 / GRP78 / HSP90 co-localized with the autophagosome vesicle protein LC3 (ATG8) after drug exposure. Treatment of GBM5 and GBM12 cells for 3 h with OSU-03012 or [OSU-03012 + sildenafil] caused phospho-ATG13 S318 or HSP27 to co-localize with LC3 in punctate bodies (Figure S8A). Treatment of GBM5 and GBM12 cells for 3 h with OSU-03012 or [OSU-03012 + sildenafil] caused GRP78 to co-localize with the autophagosome vesicle marker protein LC3 (Figure S8B). In contrast, HSP90 and LC3 did not co-associate prior to or after drug exposure. Thus we conclude it is the combined dysregulation of both GRP78 *and* HSP27 that is required for drug-induced promotion of the toxic autophagy signal.

In addition to ULK-1, another protein kinase was detected in our mass spectrometry proteomic screen; Polo-like kinase 4 (PLK4). PLK4 is localized in the nucleus where it is thought to regulate centriole biology within centrosomes during DNA synthesis. PLK4 interacts with the kinase BuBR1 which is localized in the kinetochore and BuBR1 associates with the scaffold protein BUB3 which collectively regulate the mitotic spindle checkpoint. Treatment of cells with OSU-03012 or sorafenib, with or without sildenafil, did not significantly alter PLK4, BUB3 or BuBR1 expression (data not shown).

Our screen identified several proteins involved in reactive oxygen species detoxification: peroredoxin and thioredoxin. Thioredoxin is a well-known client of HSP90 and is also known to associate with peroredoxin, presumably in a large chaperone complex [36–38]. HSP27 maintains thioredoxin reductase expression activity which is vital to maintain thioredoxin as a reactive oxygen species detoxification enzyme. Treatment of cells with drugs modestly reduced the levels of peroredoxin (Figure S9A). Treatment of cells with OSU-03012 or with sildenafil (which generates both ROS and RNS) strongly lowered thioredoxin expression. Prior studies from our group have shown that agents which quench reactive oxygen species levels, such as N-acetyl cysteine, reduce the ability of OSU-03012 to kill tumor cells. Transfection of GBM cells with a plasmid to express a wild type thioredoxin protein suppressed the induction of reactive oxygen species caused by OSU-03012 treatment, and transfection to express a mutant inactive form of the protein caused a prolonged high level of ROS (Figure S9B). Forty eight hours after drug treatment, over-expression of thioredoxin had suppressed OSU-03012 killing whereas over-expression of the mutant protein strongly promoted drug-induced cell death (Figure S9C). Over-expression of HSP27 or of GRP78, maintained thioredoxin expression in the face of both drug combination treatments (Figure S9D).

We over-expressed chaperone proteins and determined their relative impact on the ability of our drug combinations to induce tumor cell death over 24 h. Over-expression of HSP90, GRP78 or of [GRP78 + HSP27] or of [HSP70 + HSP27] significantly protected tumor cells from exposure to the toxic drug combinations (Figure S10A). Over-expression of all chaperones, alone or in combination, to some extent maintained expression of c-FLIP-s, MCL-1 and TRX after drug combination exposure (Figure S10B). Thus by inhibiting the functions of multiple chaperones our drug combinations reduce the expression of multiple cyto-protective proteins (TRX, c-FLIP-s, MCL-1, BCL-XL); facilitate the inactivation of cyto-protective signaling pathways (mTOR); and facilitate the activation of cyto-toxic pathways (PERK-eIF2α).

## DISCUSSION

At present OSU-03012 (AR-12) has obtained the orphan designation for cryptoccosis and tularaemia in Europe, and the licensee of OSU-03012, Arno Therapeutics, has entered into a cooperative research and development agreement with the US Army Medical Research Institute of Infectious Diseases for AR-12. Ourselves and others have shown that OSU-03012 (AR-12) has anti-tumor activity *in vitro* and *in vivo* in many model systems as well as having anti-viral and anti-bacterial properties against organisms as diverse as Mumps, Measles, Influenza, Ebola, Marburg, and pan-antibiotic resistant strains of *Neisseria gonorrhea* and *Klebsiella pneumoniae*. Indeed, over the last 10 years we have published multiple manuscripts detailing the cellular targets of OSU-03012 (AR-12). The “big-picture” conclusions we had previously made concerning the biology of the drug prior to the present manuscript were that OSU-03012 (AR-12) kills tumor cells selectively over non-transformed cells and does so by primarily inactivating and down-regulating the chaperone GRP78 which causes a prolonged intense endoplasmic reticulum stress response leading to high levels of autophagy flux, mitochondrial dysfunction and an apparent necroptotic form of tumor cell death.

The present studies extend our knowledge to encompass proteomic analyses of AR-12 interacting proteins, demonstrating that multiple other chaperones and chaperone regulatory proteins interact with the drug in a manner which permits drug-dependent precipitation of those proteins. The most notable novel chaperone found to associate with AR-12, albeit indirectly due to its lack of a purine binding domain, and explain its mechanism of action, was HSP27. AR-12 was originally proposed to exhibit anti-tumor activity through inhibition of the enzyme PDK-1 within the PI3K pathway, a mechanism which we largely disproved in 2005 and 2006. In 2014 and earlier in 2015 we proposed that one reason AR-12 may have been thought to be a PDK-1 inhibitor was because cell surface GRP78 had been shown to play an intimate role in maintaining PI3K / PDK-1/AKT activity, and we discovered that AR-12 very rapidly caused breakdown of cell surface GRP78 [39–42]. Our present data demonstrating AR-12 mediated reductions in HSP27 expression and changes in its localization, including in autophagosomes, further strengthens this argument as several groups have shown that HSP27 plays a key chaperone function in facilitating PI3K/AKT activation, which in turn provides a potent downstream anti-apoptotic signal in its own right, and through mTOR an anti-autophagy signal [43, 44]. That AR-12 targets multiple chaperone proteins whose clients play essential roles in maintaining anti-apoptotic signaling through the PI3K pathway strongly supports the use of AR-12 at the very least as a cancer therapeutic index modulator to enhance the effectiveness of standard of care therapies, and in the case of our data, glioblastoma multiforme. Prior studies from the Dent laboratory have demonstrated in PDX orthotopic animal models of GBM that OSU-03012 can prolong animal survival and radio-sensitize tumors resulting in a further prolongation of survival [6].

The role of HSP27 in the regulation of other PI3K pathway regulated processes also connects our prior studies with AR-12 to the new data in this manuscript [43–50]. As GRP78 and HSP27 both act to maintain PI3K / AKT signaling they also could be thought to very likely play a key role in the regulation of the PI3K-regulated kinase mTOR. Our present data strongly argues that regulation of mTOR function by HSP27 may be of greater importance from a therapeutic standpoint than modulation of AKT. Under conditions of nutrient surplus or growth factor stimulation mTOR is active and both enhances cell proliferation and mobility but also suppresses catabolic processes such as macro-autophagy through inhibitory phosphorylation of ULK-1 (known as ATG-1 in yeast). We noted that AR-12 reduced the phosphorylation of mTOR at S2448, to a much greater extent than that of AKT T308, indicative of reduced mTOR kinase activity, which correlated with reduced phosphorylation of the inhibitory mTOR phosphorylation site in ULK-1, Serine 757. ULK-1 activation concomitant with Serine 757 dephosphorylation was demonstrated by increased ATG13 phosphorylation at Serine 318, a site known to be targeted by ULK-1. Thus combined loss of GRP78 and HSP27 facilitates a reduction in mTOR activity which in turn promotes ULK-1–dependent ATG13 phosphorylation and hence autophagosome formation.

In previous work we had linked AR-12 effects at increasing autophagosome formation to reduced GRP78 expression and in parallel the activation of PERK and elevated endoplasmic reticulum stress signaling. In transformed rodent fibroblasts, AR-12 increased the expression of Beclin1 and LC3 in a PERK dependent manner, in general agreement with the present data in human tumor cells showing that dominant negative eIF2 alpha also suppressed AR-12–induced expression of the autophagy regulatory, and autophagy facilitating proteins, Beclin1 and LC3. Furthermore, in this manuscript we demonstrated that AR-12 causes the co-localization of both GRP78 and HSP27 with LC3 in punctate bodies i.e. autophagosomes. Thus collectively our data from previous work and the present studies argue that by modulating the function of the chaperones GRP78 and HSP27, regardless of the many other chaperones we also now know are effected by the drug, AR-12 promotes toxic autophagosome formation in tumor cells through two distinct mechanisms and thus this is why the catabolic process is associated with OSU-03012–induced *cell death*.

HSP27 biology is also regulated by protein phosphorylation, with the p38 MAPK pathway causing increased phosphorylation at Serine 15, Serine 78 and Serine 82 in the chaperone [43–50]. Others have shown these modifications lead to the basal state high molecular weight oligomer complexes of HSP27 (> 700 kDa) disassociating into low molecular weight tetramers and dimers (∼ 50 kDa) [45]. In the present studies we discovered that AR-12 caused the high molecular weight HSP27 complexes visible at 60X magnification to dissociate, which correlated with only modest levels of p38 MAPK phosphorylation and increased HSP27 phosphorylation at Serine 15, but not at Serine 78, and marginally at Serine 82 only in GBM12 cells. It is known that Serine to Aspartic acid phospho-mimicking HSP27 mutants have a decreased oligomer size, enhanced chaperone activity with in parallel an elevated binding capability of destabilized lysozyme. Very recent data from Jovcevski et al. has also demonstrated that the HSP27-S15D and HSP27-S78D/S82D proteins form large disperse complexes when compared with wild type HSP27, with a shift toward smaller oligomeric species [45]. The chaperoning activity of all single HSP27 mutants was equivalent in the assays of Jovceski et al. as was the case for the double HSP27 mutant proteins, arguing that no single phospho-serine residue has a predominant role in regulating the chaperone activity of HSP27. i.e. the cumulative acquisition of one, then two, then three phosphorylations regulates the degree of HSP27 oligomerization. Thus through both p38 MAPK signaling, as well as through a more direct interaction through HSP70 detected through proteomics and co-immuno-precipitation, AR-12 modulates HSP27 biology.

Our data sets determining chaperone expression levels using immuno-fluorescence and western blotting were very often discordant. This was in contrast to our data with non-chaperone proteins that bound ATP and could hydrolyze ATP, i.e. ERK2 and JNK1. The expression and localization of nuclear localized PLK-4 and its chaperones was not altered by AR-12 or sorafenib. One possibility we explored for our data set differences was that upon treatment of cells with AR-12 (or sorafenib) chaperones were becoming localized inside vesicles that could not be solubilized with a 0.5% solution of Triton X100. Thus we performed studies using the complex former β-cyclodextrin, which additionally solubilizes Triton X100 resistant sphingolipid membranes, as well as with CHAPS, a detergent used when lysing cells and isolating the active conformations of the BCL-2 homology domain 3 (BH3) proteins BAX and BAK. Neither detergent altered the immuno-fluorescence staining pattern of chaperones that was observed when using Triton X100. This argues against the hypothesis that OSU-03012–induced localization changes into locations that prevent immuno-detection are occurring.

More obviously, when using antibodies raised against epitopes in different portions of HSP90, HSP70, HSP27 and GRP78 we also observed discordant data, using immuno-fluorescence detection. Antibodies raised against the ATP-binding NH_2_-terminal domains of HSP90, HSP70 and GRP78 demonstrated that OSU-03012 treatment reduced immuno-fluorescence detection whereas those antibodies raised against central and COOH-terminal epitopes remained constant or exhibited more modest declines in detection. Our data using bacterial and eukaryotic expressed chaperones also strongly argued that OSU-03012 was interacting with chaperone proteins through the ATP binding site within their ATPase domain. At this point it is also important to consider additional proteomic data, where a number of other ATP, GTP and nucleotide binding proteins were identified in our proteomic screen using OSU-03012, though notably not PDK-1 (Figure S11). Some of the proteins listed in Figure S11 are known to have chaperone partners, such as HSP90 and RAS proteins [51]. That so many proteins in the proteomic screen with AR-12 either bind ATP/GTP/nucleotides, together with our in silico docking data, tends to argue that AR-12 is able to interact with some conserved structural portion of a purine binding site *n.b.* no pyrimidine specific binding protein was identified. Thus our present findings suggest that AR-12 treatment modifies the tertiary structure of ATP binding chaperones to such an extent that NH_2_-terminal antibody recognition epitopes become lost when examining the native protein and only become available for detection again after boiling in SDS and detection by western blot.

The chemical structures of OSU-03012 (AR-12); celecoxib; sorafenib; regorafenib; pazopanib; and AR-13 are presented in Figure S12, alongside those of guanosine, adenosine, cytidine, thymidine and uridine. That the inclusion of a single fluorine atom in sorafenib, which results in the drug regorafenib, should so significantly reduce the HSP90 ATPase inhibiting effect of the drug argues that the additional fluorine atom either induces a steric clash or alters the conformation of the ring system so much as to prevent chaperone association. Our initial exploratory ATPase studies used OSU-03012, sorafenib and regorafenib. To our surprise we then discovered that pazopanib was a more potent ATPase inhibitor than OSU-03012, whereas celecoxib was found to be a very poor ATPase inhibitor. When we compared the structures of OSU-03012, its parent drug celecoxib, sorafenib and pazopanib we noted that sorafenib, OSU-03012 and pazopanib all contained complex extended long ring structures including nitrogen atoms in the ring systems, as observed for nucleotides (dashed green circles). Both pazopanib and celecoxib contain sulfone moieties (dashed red circles). Thus a priori to any experiment we predicted, based on its structure including portions of the other ATPase inhibitors that the novel compound AR-13 would be an HSP90 ATPase inhibitor. We subsequently discovered that AR-13 was significantly more potent at inhibiting chaperone ATPase activity than either pazopanib or OSU-03012. AR-13 is, in essence, the drug celecoxib with a phenanthrene group in place of the *p*-tolyl group. (dashed purple circles). As AR-13 is a significantly better HSP90 and HSP70 ATPase inhibitor than celecoxib we thus conclude that the phenanthrene system in AR-12 and in AR-13 is essential for their enhanced biological activities toward HSP90 and HSP70 chaperone ATPase activities compared to their parent compound celecoxib.

As our studies were nearing completion a molecular modeling group in India published computer generated structures of OSU-03012 interacting with the ATPase sites of GRP78 and HSP70 [52]. These studies focused on polar hydrogen bonding interactions between OSU-03012 and the chaperone proteins, ignoring any hydrophobic interaction through the phenanthrene group. Based on the modeled energies of association/dissociation of OSU-03012 with the chaperones these authors concluded that OSU-03012 would not alter the conformation of the HSP70 or GRP78 chaperone proteins. Our data examining the NH_2_, middle and COOH portions of HSP90 shows that this chaperone does alter its 3-dimensional structure, of the NH_2_-terminus, in response to OSU-03012 exposure, and our data comparing the ATPase inhibitory effects of our compounds localized the long hydrophobic three ring C6 structure as a key molecular motif for chaperone inhibition. Based on in silico docking studies we found that OSU-03012 could associate with the HSP90 hydrophobic purine pockets of these chaperones. Studies beyond the remit of this manuscript will be required to fully model the association of AR-12 with purine binding sites in chaperone and other proteins.

## MATERIALS AND METHODS

### Materials

OSU-03012, sildenafil, regorafenib, pazopanib, sorafenib tosylate were purchased from Selleckchem (Houston, TX). OSU-03012 (AR-12) and AR-13 were kindly provided by Arno Therapeutics (Flemington NJ). Cells were purchased from the ATCC and were not further validated beyond that claimed by ATCC. Cells were re-purchased every ∼6 months. Primary human glioblastoma (GBM) cells, developed by Dr. C.D. James when at the Mayo Clinic (Rochester, MN) has been described previously [6–9]. ADOR non-small cell lung cancer cells are personal a donation from the patient to the Dent laboratory. De novo cisplatin resistant “Spiky” ovarian cancer cells, a patient derived explant (PDX) model, were kindly provided by Dr. Karen Paz (Champions Oncology, NJ). The plasmid to express GRP78 was kindly provided to the Dent laboratory by Dr. A.S. Lee (University of Southern California Los Angeles, CA). The plasmids to express thioredoxin (TRX) and mutant thioredoxin (mTRX) were a kind gift from Dr. David Gius (Radiobiology Branch, National Cancer Institute, Bethesda, MD). The plasmids to express HSP27, eIF2α S51A, kinase-inactive PERK and all others listed in this manuscript were purchased from Addgene (Cambridge, MA). Commercially available validated short hairpin RNA molecules to knock down RNA/protein levels were from Qiagen (Valencia, CA) or were supplied by collaborators [12, 13].

### Methods

#### Protein detection

*Cell treatments, SDS-PAGE and Western blot analysis.* Cells were treated with various drug concentrations, as indicated in the figure legends. SDS-PAGE and immunoblotting was performed as described in references [6–9]. For SDS-PAGE and immunoblotting, cells were plated at 5 × 10^5^ cells/cm^2^ and treated with drugs at the indicated concentrations and after the indicated time of treatment, lysed in whole-cell lysis buffer (0.5 M Tris-HCl, pH 6.8, 2% SDS, 10% glycerol, 1% *β*-mercaptoethanol, 0.02% bromophenol blue), and the samples were boiled for 30 minutes. The boiled samples were loaded onto 10–14% SDS-PAGE and electrophoresis was run overnight (10–100 *μg*/lane based on the gel size). Proteins were electrophoretically transferred onto 0.22-*μm* nitrocellulose, and immunoblotted with various primary antibodies against different proteins. Antibodies used include: HSP90 (E289) (Cell Signaling); HSP90 (#2928) (Abcam); HSP90 (ab195575) Abcam; HSP90 3G3 (13495) (Abcam); GRP78 (50b12) (31772) (Cell Signaling); GRP78 (ab191023) Abcam; GRP78 (ab103336) Abcam; GRP78 (N-20) (sc-1050) Santa Cruz; HSP27 (G31) (2402P) Cell Signaling); HSP27 [EP1724Y] (ab62339) Abcam; HSP27 (H-77) (sc-9012) Santa Cruz; HSP27 (LS-C31836) Lifespan science Corp. Other antibodies were as used in prior studies by the laboratory. All immunoblots were initially visualized at 75 dpi using an Odyssey infrared imager (Li-Cor, Lincoln, NE), then processed at 9999 dpi using Adobe Photoshop CS6. For presentation, immunoblots were digitally assessed using the provided Odyssey imager software. Images have their color removed and labeled figures generated in Microsoft PowerPoint.

### Cell death measurements by live/dead assay

Cells were grown in 96-well plates with each well containing ∼10,000 cells in 200 micro-liters of media. Cells were treated with the indicated concentrations of drugs for the indicated amounts of time in each panel. Plates were then centrifuged (500 rpm, 5 min) to re-adhere floating dead cells to the base of each well. The media was removed and live/dead assay reagent added and cells incubated for 10 min before the reagent was removed. Cells were imaged in a Hermes WiScan instrument under 10× magnification. Green cells = viable; yellow/red cells = dying/dead. The numbers of viable and dead cells were counted manually from three images taken from each well combined with data from another two wells of separately treated cells (i.e. the data is the mean cell dead from 9 data points +/− SEM from three separate exposures).

### Proteomic/mass spectrometry procedures

#### Preparation of biotin-AR-12

The strategy used to covalently link biotin to AR-12 exploited structure-activity relationships established by the Chen laboratory. Briefly, a biotin-linker arm conjugate (NHS-PEG12-biotin) was added to a region of the compound that was not associated with biological activity; its relatively long length was selected to minimize potential interference with the compound's active sites.

The linker arm has this structure: 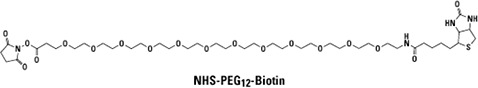


The synthetic scheme is: 
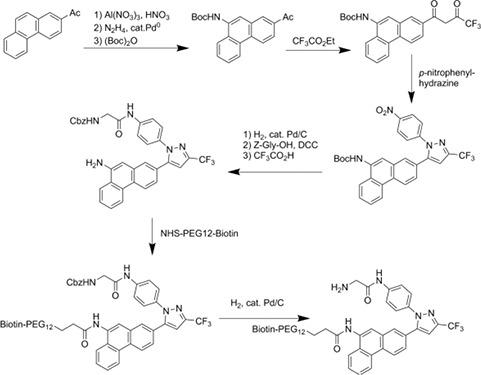


This scheme yields the following final biotin-AR-12 conjugate: 
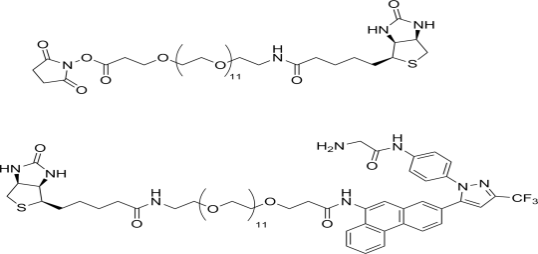


### Sample preparation, SDS-PAGE and tandem mass spectrometry analysis

Cultured SKBR3 HER2+ human mammary carcinoma cells were extracted after 24 h in serum free medium with ice cold buffer containing non-ionic detergent (Triton X100), protease and phosphatase inhibitors. Lysates were cleared of insoluble material by centrifugation. Supernatants were “pre-cleared” of molecules that bind non-specifically to streptavidin (SA) coated beads by incubating with SA-beads for 30 min at 4°C and subsequently removing the beads by centrifugation. Supernatant samples resulting from this step were then incubated with biotin-AR-12 in the presence or absence of excess AR-12 for 2 h at 4°C. SA-beads were then added for an additional 2 h at 4°C before capture by centrifugation, repeated washing with non-ionic detergent buffer and extraction with boiling Laemmli SDS-PAGE sample buffer. Samples were subjected to SDS-PAGE and bands were visualized using Ponceau S. Bands present in the biotin-AR-12 samples that were not found in the samples incubated with excess unlabeled AR-12 were excised from the gel, rinsed to remove Ponceau S and digested with trypsin. Eluted tryptic peptides were separated by nanotrap- and C18-column HPLC coupled to online analysis by tandem mass spectrometry (nLC-ESI-MS/MS) on an ion trap mass spectrometer equipped with a nanospray ion source using conventional techniques (Laboratory of Proteomic and Analytical Technologies, Frederick National Laboratory for Cancer Research, National Cancer Institute, Frederick, MD). The resulting MS/MS spectra were searched against the human IPI database to derive probable peptide amino acid sequence, protein/gene identities and statistical confidence values (http://blast.ncbi.nlm.nih.gov/Blast.cgi).

### Isolation of GST-NH_2_ terminal HSP90 from bacteria and the measurement of its ATPase activity

*E. coli* BL21 [F–, *ompT*, *hsdS* (rB, mB), *gal*] were transformed with a plasmid to express a fusion protein of the NH_2_-terminal portion of HSP90 fused to glutathione-S-transferase (GST) (purchased from Addgene). Bacteria were grown at 24°C with shaking until the A_600_ reached 0.6. IPTG was added to a final concentrationof 0.1 mM and the incubation continued for an additional 6 hours. Bacteria were recovered by centrifugation and stored on ice. The cell pellet was resuspended in 50 μl of ice-cold 1X PBS per ml of culture. The re-suspended cells were mechanically disrupted using a probe sonicator, on ice, in short 5 second bursts. Triton X-100 was added to a final concentration of 1% (v/v) followed by 30 minutes of gentle shaking to aid in solubilization of the fusion protein. Bacterial debris and denatured proteins were removed by centrifugation at 15,000 × g for 20 min at 4°C. The supernatant was removed and immediately mixed with pre-equilibrated Glutathione Sepharose 4B (2 ml of a 50% Sepharose slurry is mixed with 100 ml of clarified bacterial sonicate). The Sepharose slurry was gently rotated in a cold room for 30 min. The slurry mixture was centrifuged (10,000 × g for 10 min) and the supernatant discarded. The Glutathione Sepharose was washed with 10 bead volumes of 1X PBS. The Sepharose slurry was centrifuged (5,000 × g, 5 min), and the supernatant discarded. The Sepharose beads were washed three times. Chaperone ATPase activity using the ATPlite 1step kit (PerkinElmer) was determined using GST-NH_2_ terminal HSP90 still linked to the Glutathione Sepharose 4B beads. The Sepharose beads were equilibrated in the reaction buffer provided by the manufacturer for 30 min with gentle mixing, and the beads recovered by centrifugation. The beads were then resuspended 1:1 with reaction buffer. To each well of a 96 well plate was added 50 μl of bead slurry and 50 μl of substrate buffer solution containing vehicle control or drug to achieve the desired final drug concentrations. The reactions were started using a multi-channel pipette delivering 50 μl of reconstituted reagent to each well. The plate was placed in foil in an orbital shaker at 37°C for 15 min. The plate was removed, centrifuged to remove floating Sepharose beads, and 100 μl of the supernatant from each well placed into a new well in another 96 well plate. The light emitted from each well/treatment condition was quantified using a Vector 3 plate reader (*n* = 3 of three studies +/− SEM).

### Assessment of reactive oxygen/nitrogen species generation

GBM12 cells were transfected with empty vector (CMV) or with plasmids to express wild type thioredoxin (TRX) or a mutant inactive thioredoxin (mTRX), and 24 h after transfection cells were re-plated into 96-well plates. Cells were treated with 1 μM OSU-03012 for the indicated periods. Fifteen minutes before isolation, cells were incubated with 2′,7′-dichlorodihydrofluorescein diacetate (dihydro-DCF; Invitrogen) (5 μM), which is non-fluorescent in its dihydro form but upon reaction with reactive oxygen (ROS)/nitrogen species becomes highly fluorescent. Dihydro-DCF is sensitive to oxidation by hydroxyl radicals and peroxynitrite directly and hydrogen peroxide in the presence of a peroxidase. Fluorescence measurements were obtained 15 min after OSU-03012 addition with a Vector 3 plate reader. Data are presented corrected for baseline fluorescence of dye at each time point. Each time point represents the mean of six data points.

### Gel filtration of HeLa cell lysates

HeLa cells were grown in 25 cm^2^ flasks were exposed to AR-12 or sorafenib (both, 5 μM) or DMSO control for 20 mins. Media was removed and cells washed with PBS containing 5 μM of drug (or equivalent volume of DMSO) two times. Cells were lysed with 1% Triton X-100 in PBS containing protease and phosphatase inhibitors (Pierce) and 5 μM of drug or equivalent volume of DMSO as a control. Samples were vortexed and then incubated at 4°C for 30 mins on ice. Samples were centrifuged (20,000 × g, 20 min, 4°C) and supernatants collected and stored at 4°C until they were subjected to gel filtration. A Superdex 10/300 column (GE Healthcare) was pre-equilibrated with buffer (PBS) containing 5 μM drug or equivalent volume of DMSO at 0.5 ml/min. 200 μg of total protein was loaded onto the column (100 μl of sample) and proteins eluted with buffer at 0.5 ml/min. Fractions of 1 ml were collected as they eluted from the column. Those fractions containing protein (8–23) were collected and initially stored on ice. To each fraction, 250 μl of ice-cold trichloroacetic acid was added to a final concentration of 10% (w/v) to precipitate the proteins; each fraction was mixed vigorously and then incubated on ice overnight. Samples were then centrifuged (20,000 × g, 20 min, 4°C), the supernatant discarded and the protein pellet washed twice with cold acetone followed each time by additional centrifugations. Samples were then left to air-dry prior to SDS-PAGE and immunoblotting.

### Molecular dynamics

The model system for HSP90 was built using VMD. For the model, the coordinates of human HSP90 in complex with ACP (PDB refcode 3T10) were used. The protein was inserted into a cubic water box of size 80 * 80 * 80 Å^3^. The whole system consisted of 47971 atoms. For MD simulations, NAMD 2.10 was used. Ligand and protein were restrained to their original positions and the system was equilibrated at constant temperature and pressure (300 K, 1 atm, NPT ensemble) for 0.5 ns. Simulations were done using the CHARMM36 force-field in combination with the CGenFF forcefield where needed. Then 5 ns of production MD was performed. Docking (Autodock Vina) was done against conformations extracted from this run. Initial CGenFF-compatible parameters for the AR-12 were derived using the CGenFF website and subsequently optimized. Subsequently, 50 ns of accelerated MD with Generalized Born Implicit Solvent (GBIS) for both HSP90 in complex with AR-12 and apo-HSP90 was performed [25–41].

### Data analysis

Comparison of the effects of various treatments was performed using one way analysis of variance followed by a two tailed Student's *t*-test. Experiments were performed in triplicate and were performed individually three times (± SEM). Differences with a *p*-value of < 0.05 were considered to be statistically significant.

## SUPPLEMENTARY MATERIALS FIGURES



## Abbreviations

OSU: OSU-03012 also called AR-12
SIL: sildenafil also called Viagra
TAD: tadalafil also called Cialis
PTEN: Phosphatase and tensin homolog
R: receptor
dn: dominant negative
CMV: empty vector control plasmid
COX: cyclooxygenase
P: phospho-
ca: constitutively active
WT: wild type
PERK: PKR like endoplasmic reticulum kinase
HSP: heat shock protein
GRP: glucose regulated protein
CHAPS: 3-[(3-Cholamidopropyl)dimethylammonio]-1-propanesulfonate
