# Supplementary material for: Multi-kinase inhibitors can associate with heat shock proteins through their NH2-termini by which they suppress chaperone function
Source: Oncotarget. 2016 Feb 12;7(11):12975–96. doi: 10.18632/oncotarget.7349 (PMC4914336; doi:10.18632/oncotarget.7349)
Supplement: Supplementary file 1 [file oncotarget-07-12975-s001.pdf]

Multi-kinase inhibitors can associate with heat shock proteins through their NH_2-termini by which they suppress chaperone function

Supplementary Materials

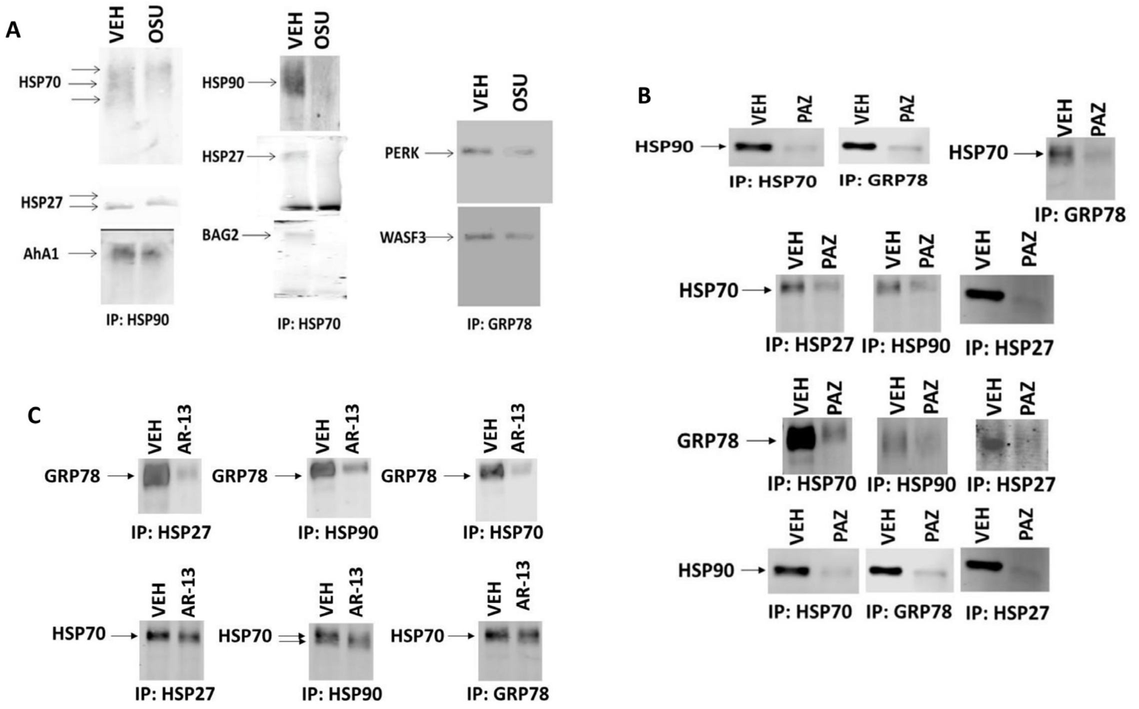

Supplementary Figure S1: OSU-03012, sorafenib, pazopanib and AR-13 alter chaperone – chaperone interactions assessed after immuno-precipitation. (**A–C**) GBM12 cells were treated with vehicle or with OSU-03012 (2 μM); sorafenib (2 μM); pazopanib (2 μM); for 10 min and then lysed. Immuno-precipitation of HSP90, GRP78, HSP27 and HSP70 was performed in the presence or absence of each drug individually (2 μM) for 3 h. Cells were washed with lysis buffer in the presence or absence of each drug individually (3 × 1 h, each). Immuno-precipitates were then boiled in SDS PAGE buffer containing glycerol and bromophenol blue. Proteins were separated on SDS PAGE (12% and 14% gels) and immunoblotting performed for the proteins as indicated.

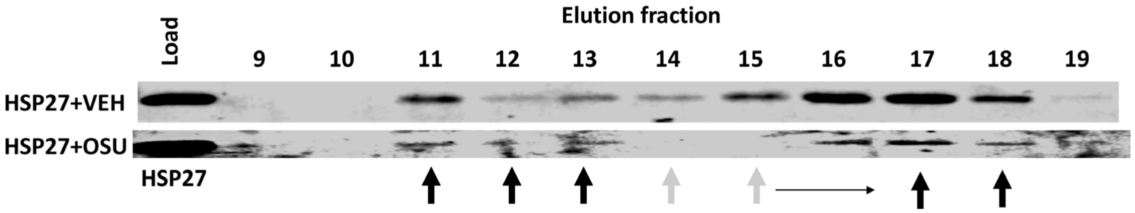

Supplementary Figure S2: Chaperone – protein interactions are regulated by OSU-03012. HeLa cells were pre-treated for 20 min with vehicle control (DMSO) or 5 μM OSU-03012 then lysed, and clarified lysates subjected to gel filtration on a Superdex column in the presence of the drug. Fractions of 1.0 ml were taken (15 fractions) and the protein precipitated from the fractions on ice using 10% (w/v) trichloro-acetic acid. Recovered protein was washed with cold 100% acetone. The protein pellets were boiled in SDS PAGE sample buffer containing bromophenol blue and subjected to SDS PAGE on 12% acrylamide gels, followed by western blotting to determine the elution profiles of HSP27.

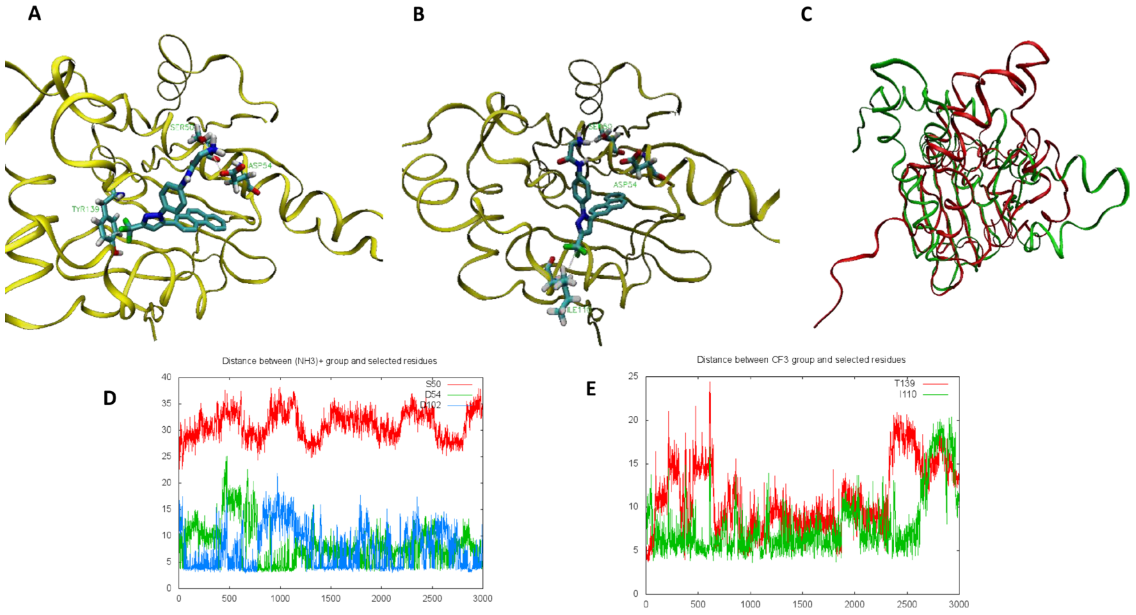

Supplemantary Figure S3: *In silico* docking of OSU-03012 with the NH2-terminal ATP binding domain of HSP90. (**A**) AR-12 docked into HSP90, first pose. (**B**) AR-12 docked into HSP90, second pose. (**C**) HSP90 in complex with AR12 (red) and apo-Hsp90 (green) after 30 ns of accelerated MD. The two poses are discussed in the results section - in both cases the amino group interacts in hydrogen bonding with S50 or D54 (or both). In the first pose, the CF3-group is engaged in hydrophobic interaction with Y139, and in the second one it is I110. Panel C. is free HSP90 and HSP90 in complex with AR-12 after 30 ns of accelerated MD. From the simulation it appears that the position of the alpha-helices are different, but the coordinates of the beta-sheets are stable. (**D**) Distances between amino group of AR-12 and residues S50, D54 and D102 of HSP90. (**E**) Distances between trifluoromethyl group of AR-12 and residues I110 and Y138 of HSP90.

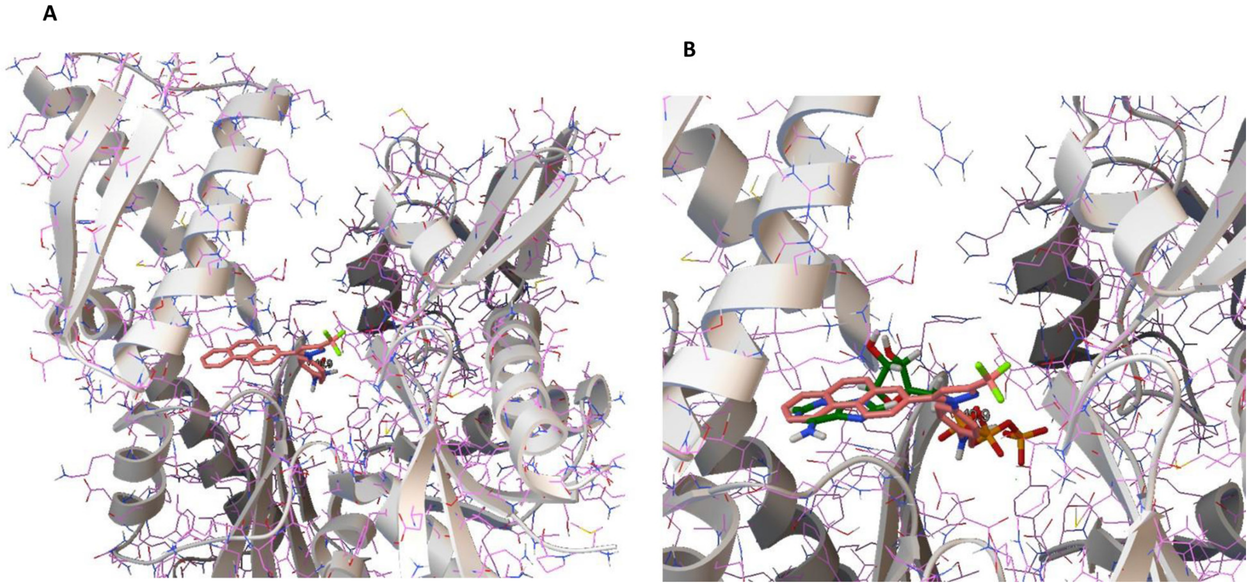

Supplementary Figure S4: *In silico* docking of OSU-03012 with the NH2-terminal ATP binding domain of HSP70. (**A**) AR-12 docked into HSP70, first pose. (**B**) HSP70 in complex with AR12 (red) and apo-Hsp90 (green) after 30 ns of accelerated MD. The most plausible AR-12 pose is attached; Figure S4A and is just from the docking. The data presented in Figure S4B has the ATP super-imposed. The phenanthrene group of OSU-03012 is in the nucleotide binding pocked (the arginine residues in this pocket may form cation-pi interactions), the -CF3 group seems to form hydrophobic interactions with one of the two tyrosines nearby, and the amino group in the side-chain interacts with the serines that would bind to the phosphate groups of ATP.

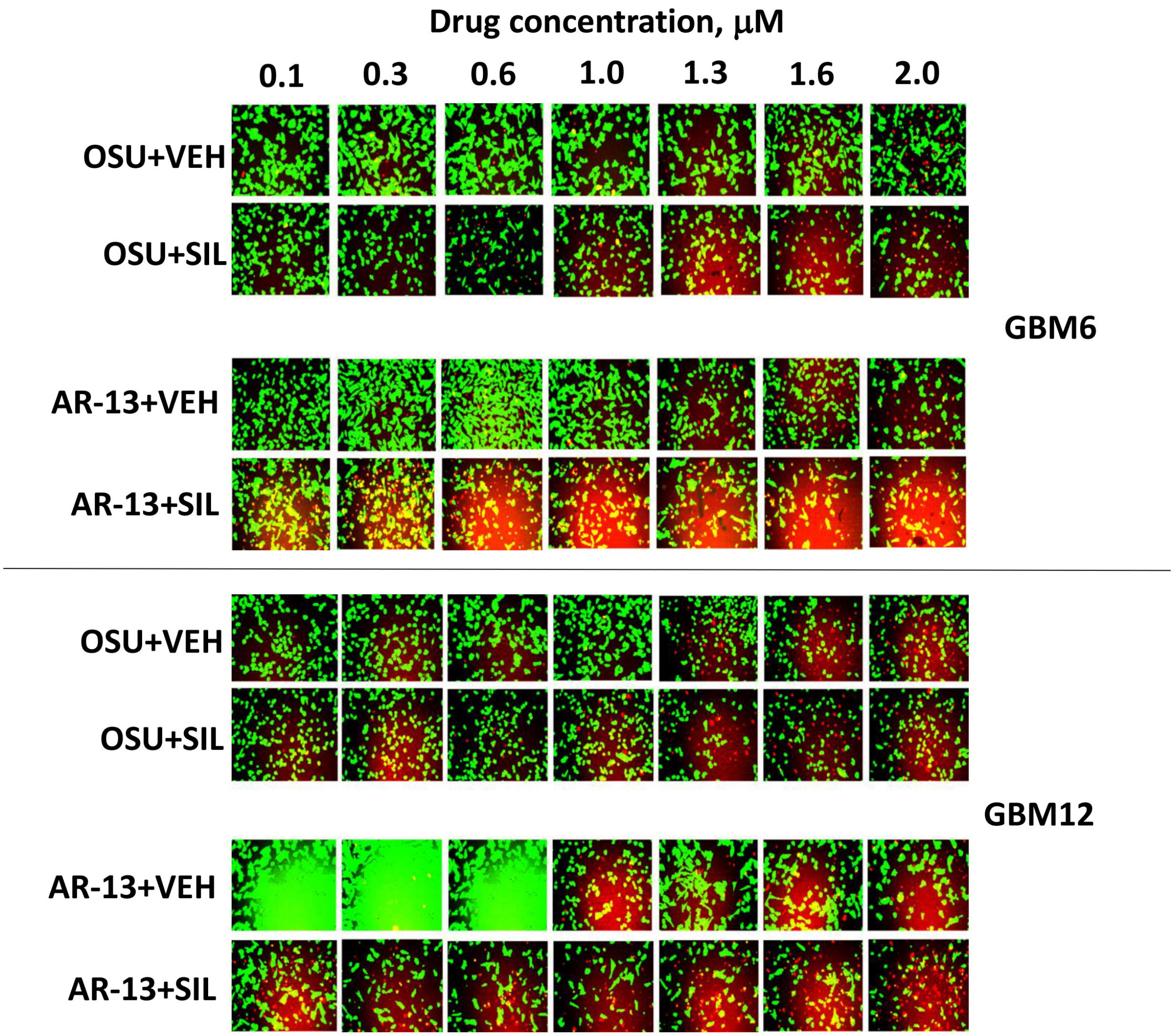

Supplemantary Figure S5: AR-13 is more potent at killing glioblastoma cells than OSU-03012 (AR-12). GBM6 and GBM12 cells were treated with increasing concentrations of vehicle, OSU-03012 or AR-13 as indicated in the presence or absence of sildenafil (2 μM). Twenty four h after drug treatments, the percentage cell death was determined at 10X magnification in a Hermes WiScan system by live/dead assay (n = 3 +/− SEM).

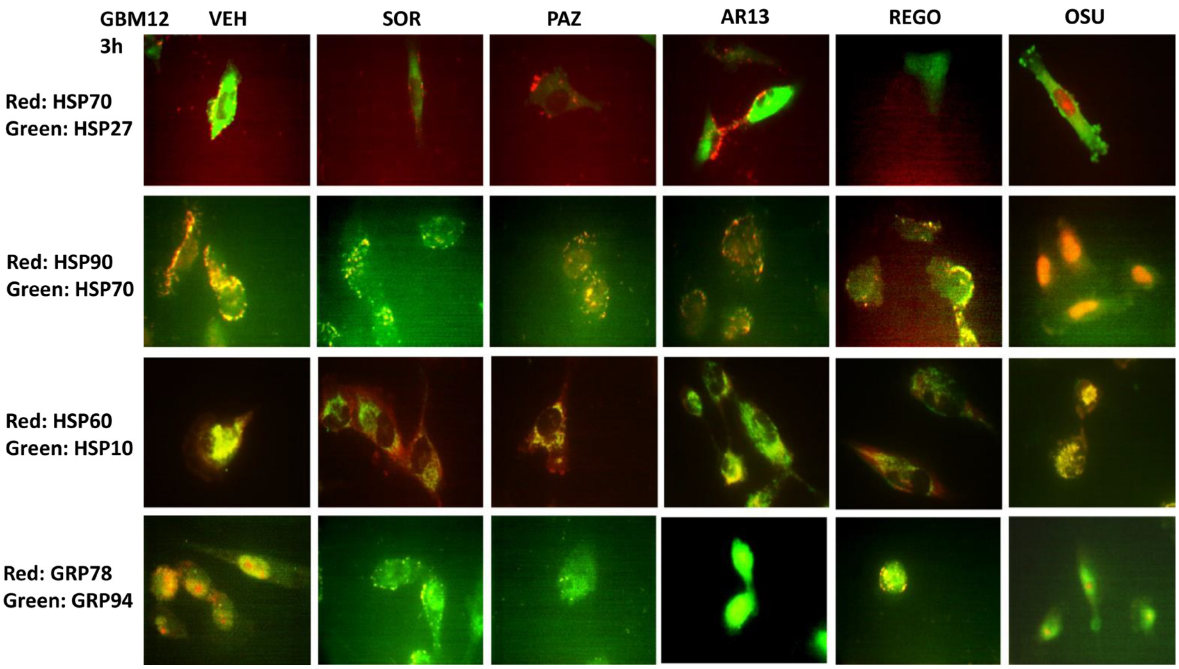

Supplamantary Figure S6: Kinase inhibitors disrupt chaperone – chaperone interactions in cells. GBM5 cells were treated with vehicle control or with: regorafenib (1 μM); sorafenib (1 μM); pazopanib (1 μM); AR-12 (OSU-03012) (1 μM); or AR-13 (1 μM) for 3 h. Cells were fixed in place and permeabilized using 0.5% Triton X100. Immuno-fluorescence was performed to detect the expression of the indicated chaperones pair-wise. Images were merged in Adobe Photoshop to indicate areas of co-localization (red + green = yellow).

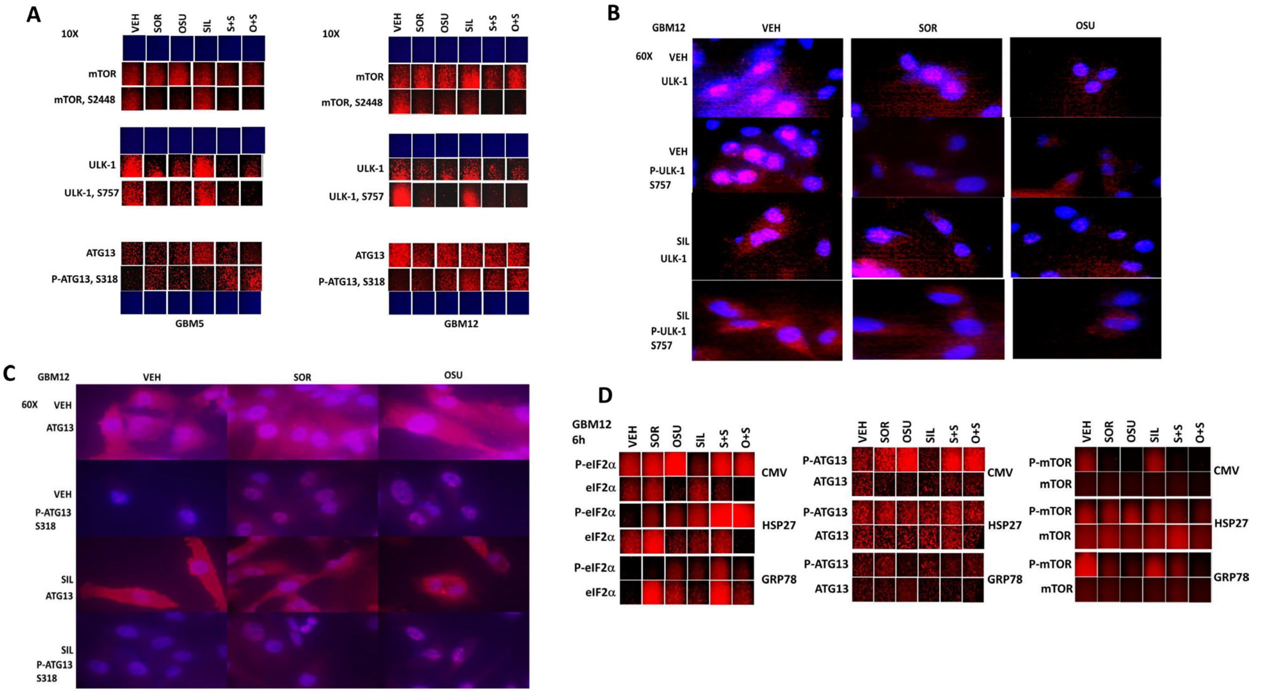

Supplamantary Figure S7: OSU-03012 and sorafenib regulate the phosphorylation and total protein expression levels of the autophagy regulatory proteins mTOR, ULK-1 and ATG13. (**A**) GBM5 and GBM12 cells were treated with vehicle, OSU-03012 (2.0 μM)/sorafenib (2.0 μM) and/or sildenafil (2 μM) for 6 h after which cells were fixed in place and permeabilized using 0.5% Triton X100. Immuno-fluorescence was performed to detect the expression levels of mTOR; ULK-1 and ATG13 as well as the phosphorylation of mTOR S2448; ULK-1 S757; ATG13 S318, presented at 10X magnification. (**B** and **C**) GBM12 cells from Panel A. were re-examined at 60X magnification to determine the localization of protein and localization of phosphorylated ULK-1 and ATG13 proteins. (**D**) GBM12 cells were transfected with empty vector (CMV), or plasmids to express GRP78 or HSP27. Twenty four h after transfection cells were treated with vehicle, OSU-03012 (2.0 μM) / sorafenib (2.0 μM) and/or sildenafil (2 μM) for 6 h after which cells were fixed in place and permeabilized using 0.5% Triton X100. Immuno-fluorescence was performed to detect the total expression levels of mTOR; eIF2α and ATG13 as well as the phosphorylation of mTOR S2448; eIF2α S51; ATG13 S318, presented at 10X magnification.

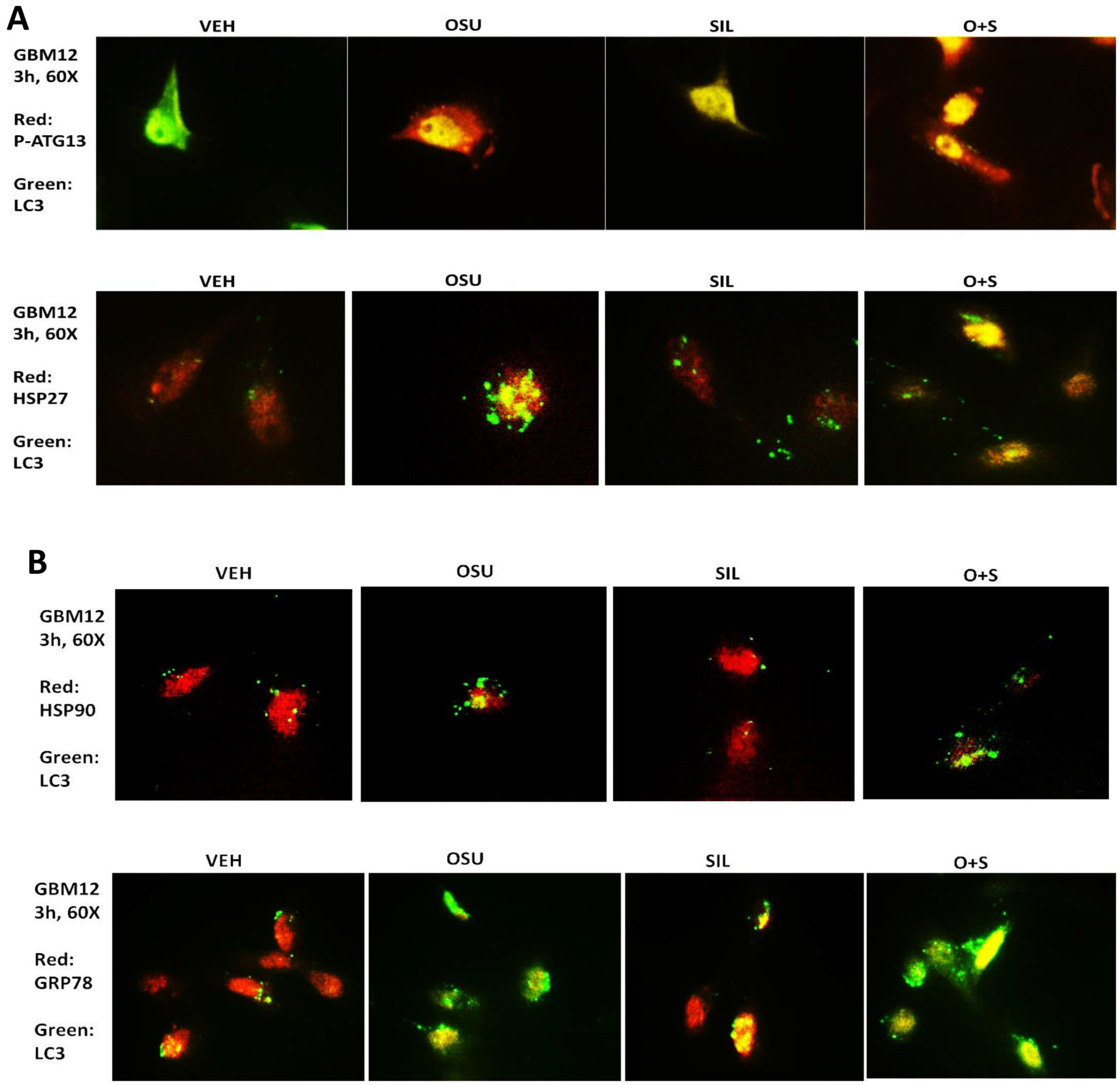

Supplemantary Figure S8: GRP78, HSP27 and phospho-ATG13 co-localize with LC3 after OSU-03012 treatment. (**A**) GBM12 cells were treated with vehicle, OSU-03012 (2.0 μM) and/or sildenafil (2 μM) for 3 h. Cells were fixed in place and permeabilized using 0.5% Triton X100. Immuno-fluorescence was performed at 60X magnification to detect the co-localization of HSP27 with LC3 and of phospho-ATG13 S318 and LC3. (**B**) GBM12 cells were treated with vehicle, OSU-03012 (2.0 μM) and/or sildenafil (2 μM) for 3 h. Cells were fixed in place and permeabilized using 0.5% Triton X100. Immuno-fluorescence was performed at 60X magnification to detect the co-localization of GRP78 with LC3 and of HSP90 and LC3.

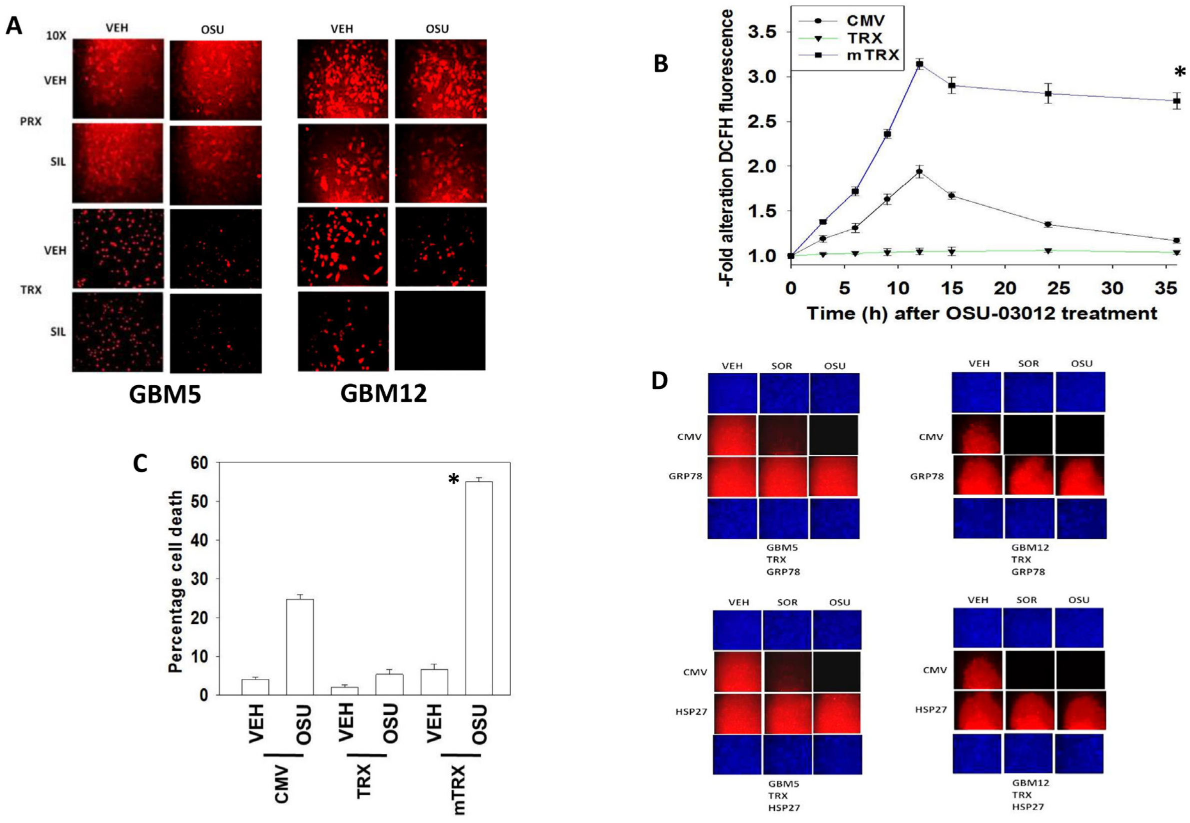

Supplemantary Figure S9: Reduced thioredoxin (TRX) expression caused by OSU-03012 results in elevated levels of reactive oxygen species and increased amounts of tumor cell death. (**A**) GBM5 and GBM12 cells were treated with vehicle, OSU-03012 (2.0 μM) / sorafenib (2.0 μM) and/or sildenafil (2 μM) for 6 h after which cells were fixed in place and permeabilized using 0.5% Triton X100. Immuno-fluorescence was performed to detect the expression levels of peroxiredoxin (PRX) and thioredoxin (TRX). (**B**) GBM6 cells were transfected with an empty vector plasmid, a plasmid to express TRX, or a plasmid to express a mutant inactive form of TRX. Twenty four h after transfection cells were treated with OSU-03012 (3 μM). At the indicated time points cells were incubated with DCFH to 10 min and then the levels of fluorescence, indicative of reactive species generation, were assessed in a Vector 3 plate reader (n = 6 +/− SEM). (**C**) GBM6 cells were transfected with an empty vector plasmid, a plasmid to express TRX, or a plasmid to express a mutant inactive form of TRX. Twenty four h after transfection cells were treated with OSU-03012 (3 μM). Twenty four h after drug exposure cell viability was determined by trypan blue exclusion assay (n = 3 +/−SEM). (**D**) Cells were transfected with an empty vector plasmid or plasmids to express GRP78 or HSP27, as indicated. Twenty four h after transfection cells were treated with vehicle, OSU-03012 (2.0 μM) / sorafenib (2.0 μM) and/or sildenafil (2 μM) for 6 h after which cells were fixed in place and permeabilized using 0.5% Triton X100. Immuno-fluorescence was performed to detect the expression levels of TRX.

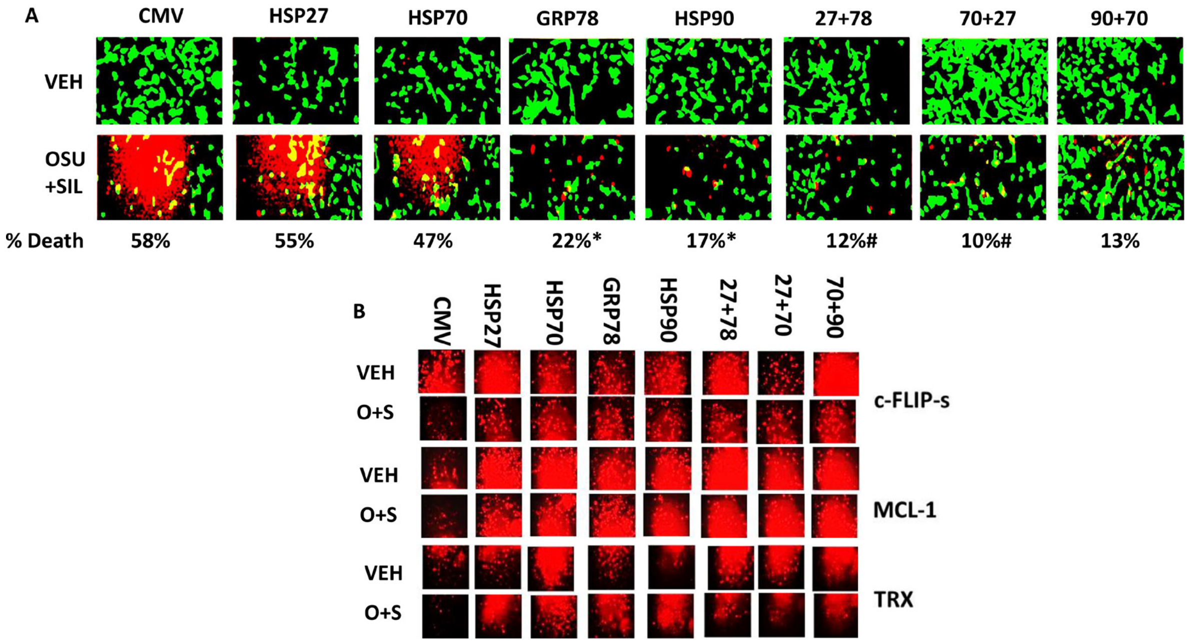

Supplemantary Figure S10: Over-expression of chaperones prevents drug combination killing. (**A**) GBM12 cells were transfected with an empty vector plasmid or with plasmids to express HSP90, HSP70, GRP78, HSP27 or in the combinations indicated. Twenty four h after transfection cells were treated with vehicle control or with: [OSU-03012 (1 µM) + sildenafil (2 µM)]; [sorafenib (2 µM) + sildenafil (2 µM)]; [pazopanib (2 µM) + sildenafil (2 µM)]; [pemetrexed (1 µM) + sorafenib (2 µM)]; ruxolitinib (5 µM) + afatinib (1 µM)]. Twenty four h after drug exposure, cells were treated with live/dead reagent and cells examined using a Hermes WiScan microscope at 10X magnification where red/yellow cells = dead; green cells = alive. (**B**) GBM12 cells were transfected with an empty vector plasmid or with plasmids to express HSP90, HSP70, GRP78, HSP27 or in the combinations indicated. Twenty four h after transfection cells were treated with vehicle control or with: [OSU-03012 (1 µM) + sildenafil (2 µM)]; [sorafenib (2 µM) + sildenafil (2 µM)]; [pazopanib (2 µM) + sildenafil (2 µM)]. Six h after drug exposure, cells were fixed in place and permeabilized using 0.5% Triton X100. Immuno-fluorescence was performed to detect the expression levels of the indicated proteins.

Additional Proteins that interact with OSU-03012 as judged using mass spectrometry.

GTPase H-Ras
Rho guanine nucleotide exchange factor 16
Elongation factor 1-alpha 1

GTP binding Proteins.

Vesicle-fusing ATPase, NSF
V-type proton ATPase catalytic subunit A, ATP6V1A
Putative ATP-binding cassette sub-family C member 13, ABCC13
ATP-dependent RNA helicase, DDX3X
Werner syndrome ATP-dependent helicase, WRN

ATP binding Proteins.

DNA-directed RNA polymerase, mitochondrial
Mismatch repair endonuclease, PMS2
DNA ligase 1, LIG1
RNA polymerase II elongation factor, ELL2
Ribonucleoside-diphosphate reductase large subunit

Nucleotide Binding Proteins.

Lysosomal acid phosphatase, ACP2
Protein phosphatase 1 regulatory subunit 36, PPP1R36
Protein phosphatase 1 regulatory subunit 12B, PPP1R12B

Phosphatases

Annexin A1, ANXA1
Annexin A2, ANXA2
Calcium/calmodulin-dependent protein kinase type II subunit gamma, CAMK2G

Calcium binding Proteins.

Supplementary Figure S11: Additional proteomic and cell biology analyses of OSU-03012 (AR-12) interacting proteins. As described in the Methods, AR-12 conjugated via biotin to sepharose beads was used to capture proteins from a whole cell lysate and those proteins specifically associating with beads in an AR-12 –dependent fashion were determined after proteolytic digestion in a mass spectrometer.

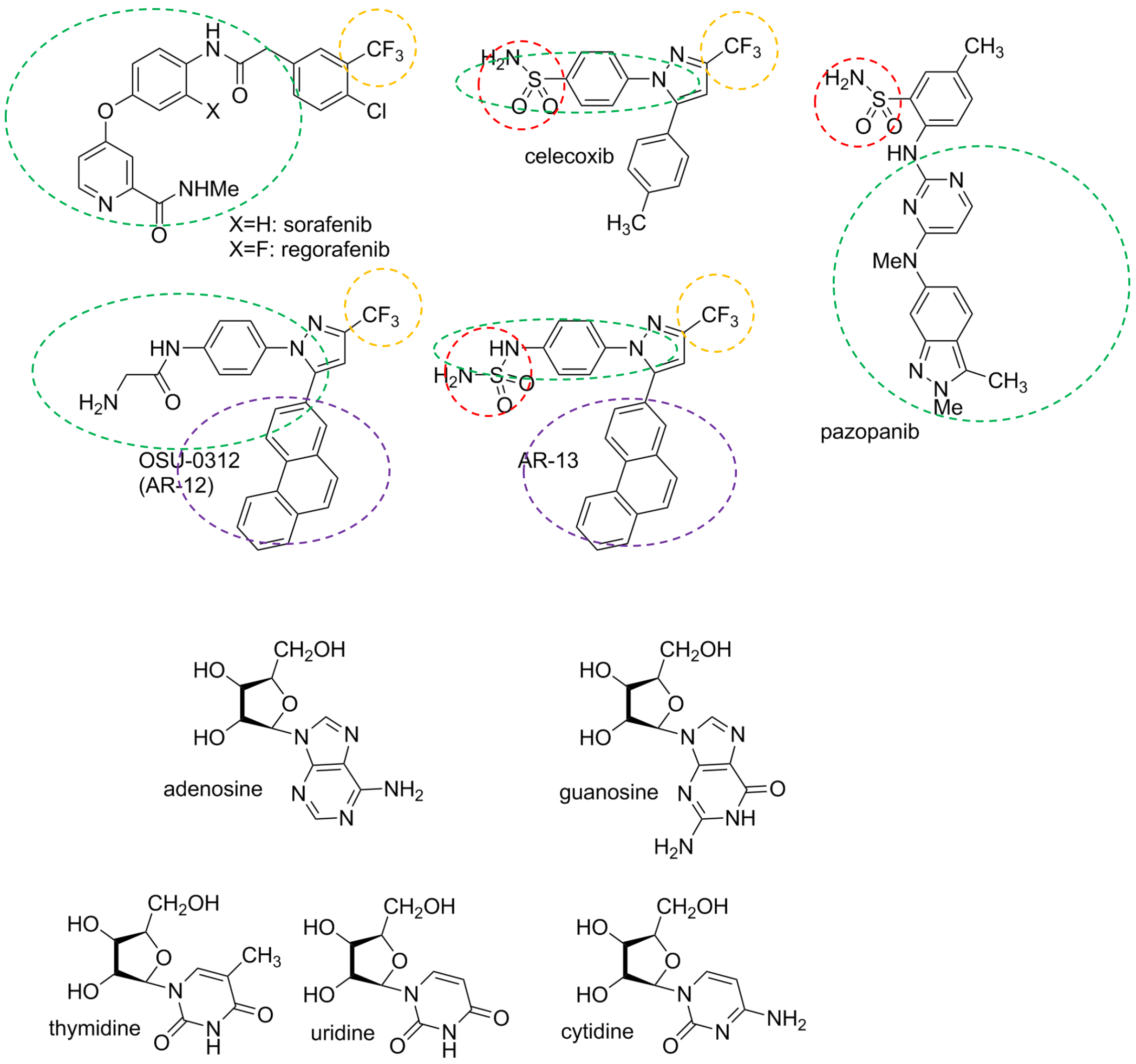

Supplemantary Figure S12: Structural comparisons between celecoxib, AR-12, AR-13, sorafenib, regorafenib and pazopanib, and adenosine; guanosine; cytidine; thymidine and uridine.
